# Aberrant Mucin Assembly in Mice Causes Endoplasmic Reticulum Stress and Spontaneous Inflammation Resembling Ulcerative Colitis

**DOI:** 10.1371/journal.pmed.0050054

**Published:** 2008-03-04

**Authors:** Chad K Heazlewood, Matthew C Cook, Rajaraman Eri, Gareth R Price, Sharyn B Tauro, Douglas Taupin, David J Thornton, Chin Wen Png, Tanya L Crockford, Richard J Cornall, Rachel Adams, Masato Kato, Keats A Nelms, Nancy A Hong, Timothy H. J Florin, Christopher C Goodnow, Michael A McGuckin

**Affiliations:** 1 Mucin and IBD Research Teams, Mucosal Diseases Program, Mater Medical Research Institute, and the University of Queensland, Aubigny Place, Mater Health Services, South Brisbane, Queensland, Australia; 2 Immunology and Inflammation Group, Phenomix Australia, Acton, Australia; 3 Molecular Genetics Team, Mater Medical Research Institute, and the University of Queensland, Aubigny Place, Mater Health Services, South Brisbane, Queensland, Australia; 4 Gastroenterology Unit, Canberra Hospital, Woden, Australia; 5 Wellcome Trust Centre for Cell Matrix Research, Faculty of Life Sciences, University of Manchester, Manchester, United Kingdom; 6 Nuffield Dept of Clinical Medicine, University of Oxford, John Radcliffe Hospital, Oxford, United Kingdom; 7 Dendritic Cell Program, Mater Medical Research Institute, Aubigny Place, Mater Health Services, South Brisbane, Queensland, Australia; 8 Phenomix Corporation, San Diego, California, United States of America; 9 Division of Immunology and Genetics and Australian Phenomics Facility, John Curtin School of Medical Research, The Australian National University, Canberra, Australia; University Hospital Schleswig Holstein, Germany

## Abstract

**Background:**

MUC2 mucin produced by intestinal goblet cells is the major component of the intestinal mucus barrier. The inflammatory bowel disease ulcerative colitis is characterized by depleted goblet cells and a reduced mucus layer, but the aetiology remains obscure. In this study we used random mutagenesis to produce two murine models of inflammatory bowel disease, characterised the basis and nature of the inflammation in these mice, and compared the pathology with human ulcerative colitis.

**Methods and Findings:**

By murine *N*-ethyl-*N*-nitrosourea mutagenesis we identified two distinct noncomplementing missense mutations in *Muc2* causing an ulcerative colitis-like phenotype. 100% of mice of both strains developed mild spontaneous distal intestinal inflammation by 6 wk (histological colitis scores versus wild-type mice, *p* < 0.01) and chronic diarrhoea. Monitoring over 300 mice of each strain demonstrated that 25% and 40% of each strain, respectively, developed severe clinical signs of colitis by age 1 y. Mutant mice showed aberrant Muc2 biosynthesis, less stored mucin in goblet cells, a diminished mucus barrier, and increased susceptibility to colitis induced by a luminal toxin. Enhanced local production of IL-1β, TNF-α, and IFN-γ was seen in the distal colon, and intestinal permeability increased 2-fold. The number of leukocytes within mesenteric lymph nodes increased 5-fold and leukocytes cultured in vitro produced more Th1 and Th2 cytokines (IFN-γ, TNF-α, and IL-13). This pathology was accompanied by accumulation of the Muc2 precursor and ultrastructural and biochemical evidence of endoplasmic reticulum (ER) stress in goblet cells, activation of the unfolded protein response, and altered intestinal expression of genes involved in ER stress, inflammation, apoptosis, and wound repair. Expression of mutated Muc2 oligomerisation domains in vitro demonstrated that aberrant Muc2 oligomerisation underlies the ER stress. In human ulcerative colitis we demonstrate similar accumulation of nonglycosylated MUC2 precursor in goblet cells together with ultrastructural and biochemical evidence of ER stress even in noninflamed intestinal tissue. Although our study demonstrates that mucin misfolding and ER stress initiate colitis in mice, it does not ascertain the genetic or environmental drivers of ER stress in human colitis.

**Conclusions:**

Characterisation of the mouse models we created and comparison with human disease suggest that ER stress-related mucin depletion could be a fundamental component of the pathogenesis of human colitis and that clinical studies combining genetics, ER stress-related pathology and relevant environmental epidemiology are warranted.

## Abstract 2

Michael McGuckin and colleagues identify two mutations that cause aberrant mucin oligomerization in mice. The resulting phenotype, including endoplasmic reticulum stress, resembles clinical and pathologic features of human ulcerative colitis.

## Editors' Summary

**Background.:**

Inflammatory bowel diseases (IBD) are common disorders in which parts of the digestive tract become inflamed. The two main types of IBD are Crohn's disease, which mainly affects the small bowel, and ulcerative colitis (UC), which mainly affects the large bowel (colon). Both types tend to run in families and usually develop between 15 and 35 years old. Their symptoms include diarrhea, abdominal cramps, and unintentional weight loss. These symptoms can vary in severity, can be chronic (persistent) or intermittent, and may start gradually or suddenly. There is no cure for IBD (except removal of the affected part of the digestive tract), but drugs that modulate the immune system (for example, corticosteroids) or that inhibit “proinflammatory cytokines” (proteins made by the immune system that stimulate inflammation) can sometimes help.

**Why Was This Study Done?:**

Although the clinical and pathological (disease-associated) features of Crohn's disease and UC are somewhat different, both disorders are probably caused by an immune system imbalance. Normally, the immune system protects the body from potentially harmful microbes in the gut but does not react to the many harmless bacteria that live there or to the food that passes along the digestive tract. In IBD, the immune system becomes overactive for unknown reasons, and lymphocytes (immune system cells) accumulate in the lining of the bowel and cause inflammation. In this study, the researchers use a technique called random mutagenesis (the random introduction of small changes, called mutations, into the genes of an organism using a chemical that damages DNA) to develop two mouse models that resemble human UC and that throw new light on to how this disorder develops.

**What Did the Researchers Do and Find?:**

The researchers establish two mutant mouse strains*—Winnie* and *Eeyore* mice—that develop mild spontaneous inflammation of the colon and chronic diarrhea and that have more proinflammatory cytokines and more lymphocytes in their colons than normal mice. 25% and 40% of the *Winnie* and *Eeyore* mice, respectively, have severe clinical signs of colitis by 1 year of age. Both strains have a mutation in the *Muc2* gene, which codes for MUC2 mucin, the main protein in mucus. This viscous substance (which coats the inside of the intestine) is produced by and stored in intestinal “goblet” cells. Mucus helps to maintain the intestine's immunological balance but is depleted in UC. The researchers show that the manufacture and assembly of Muc2 molecules is abnormal in *Winnie* and *Eeyore* mice, that less mucin is stored in their goblet cells than in normal mice, and that their intestinal mucus barrier is reduced. In addition, an incompletely assembled version of the molecule, called Muc2 precursor, accumulates in the endoplasmic reticulum (ER; the cellular apparatus that prepares newly manufactured proteins for release) of goblet cells, leading to overload with abnormal protein and causing a state of cellular distress known as the “ER stress response.” Finally, the researchers report that MUC2 precursor also accumulates in the goblet cells of people with UC and that even the noninflamed intestinal tissue of these patients shows signs of ER stress.

**What Do These Findings Mean?:**

These findings indicate that mucin abnormalities and ER stress can initiate colitis in mice. Results from animal studies do not always reflect what happens in people, but these findings, together with those from the small study in humans, suggest that ER stress-related mucin depletion could be a component in the development of human colitis. The results do not identify the genetic changes and/or environmental factors that might trigger ER stress in human colitis, but suggest that once initiated, ER stress might interfere with MUC2 production, which would lead to a diminished mucus barrier, expose the lining of the intestine to more toxins and foreign substances, and trigger local mucosal inflammation. The release of inflammatory cytokines would then damage the intestine's lining and exacerbate ER stress, thus setting up a cycle of intestinal damage and inflammation. Clinical studies to look for genetic changes and environmental factors capable of triggering ER stress and for ER-stress related changes in human UC should now be undertaken to test this hypothesis.

**Additional Information.:**

Please access these Web sites via the online version of this summary at http://dx.doi.org/10.1371/journal.pmed.0050054.

- The MedlinePlus Encyclopedia has pages on Crohn's disease and on ulcerative colitis (in English and Spanish)
- The US National Institute of Diabetes and Digestive and Kidney Diseases provides information on Crohn's disease and ulcerative colitis
- Information and support for patients with inflammatory bowel disease and their caregivers is provided by the Crohn's and Colitis Foundation of America and by the UK National Association for Colitis and Crohn's Disease
- Wikipedia has pages on mucins and on mucus (note that Wikipedia is a free online encyclopedia that anyone can edit; available in several languages)

## Introduction

Intestinal goblet cells produce the viscous mucus layer covering the intestinal epithelium. In the large intestine mucus completely fills the crypts and forms a thick coating over the mucosal surface. MUC2 mucin is the major macromolecular constituent of intestinal mucus, and is responsible for its high viscosity and forming a protease-resistant matrix that retains molecules vital to host defence. MUC2 contains a large central O-glycosylated domain and N- and C-terminal cysteine-rich domains that homo-oligomerise [1], forming a complex molecular lattice [2,3]. MUC2 is N-glycosylated in the ER, where initial homo-oligomerisation occurs [4], and the complex then moves into the Golgi apparatus where O-glycosylation, the final stages of oligomerisation, and packaging into granules occur [5]. Mucins stored intracytoplasmically in granules form the characteristic goblet cell theca from where they are secreted constitutively and in response to stimuli.

Inflammatory bowel diseases (IBD), characterized by chronic or recurrent relapsing gastrointestinal inflammation, are broadly grouped by clinical and pathological features into two groups—Crohn's disease and ulcerative colitis (UC) [6–8]. Evidence from animal models indicates that failure to suppress immunity to the abundant intestinal foreign antigen load can cause inflammation. Maintaining the normal balance between competence to respond to intestinal pathogens while not generating an inflammatory response to commensals appears to depend on the integrity of the mucosal and epithelial barriers, proinflammatory signalling pathways (especially via NF-κB), and regulation of innate and adaptive immune responses in the intestine and draining lymphoid organs. Disruption of any of these components has been shown to result in intestinal inflammation in animal models [9,10]. Defects in these components have been implicated in human IBD, although fundamental knowledge of underlying pathogenesis remains very poorly understood, with even the most well-established defect, *CARD15* mutations, elucidating only a subset of white patients with ileal Crohn's disease [9,10], and with a penetrance less than 4%. Furthermore, the pathogenic pathways that distinguish UC from Crohn's disease remain obscure.

Goblet cell and secreted mucus phenotypes represent important differences between the two diseases: in Crohn's disease, there is typically an increase in goblet cells and a thicker mucus layer [11,12], whereas in UC there is a reduction in goblet cells, decreases in MUC2 production [13,14] and sulfation [14–16], accumulation of MUC2 precursor [16], and a reduction in secreted mucus. Although it remains unclear whether changes in mucus are causative or secondary to inflammation [17], the lack of these changes in Crohn's disease indicate they are not a universal consequence of intestinal inflammation. Furthermore, a reduction in a specific biochemical fraction of colonic mucin has been described in UC patients and in their unaffected monozygotic twins [18,19], and engineered changes in intestinal mucin glycosylation result in enhanced susceptibility to toxin-induced colitis [20], raising the possibility that genetic defects in mucin physiology or biochemistry predispose to UC. On the other hand, in *Muc2* ^−/−^ mice the colitis phenotype appears to arise only on a permissive genetic background [21,22], and transgenic depletion of goblet cells not only does not trigger spontaneous inflammation, it unexpectedly reduces dextran sodium sulphate (DSS)-induced colonic injury [23]. Thus, a causal relationship between mucin abundance and colitis remains to be defined.

In this study we used random mutagenesis to produce murine models of inflammatory bowel disease. Two models were produced and we sought to characterize the basis for the pathology and identify similarities between these models and human IBD, particularly UC.

## Materials and Methods

### Generation of Mice by *N*-Ethyl-*N*-Nitrosourea Mutagenesis

All animal experimentation was approved by either the Australian National University or University of Queensland Animal Experimentation Ethics Committees. Mice were generated and housed in a PC2 specific pathogen-free facility and fed autoclaved food and water. For mutagenesis, we treated 8- to 15-wk-old male C57BL/6 mice three times at weekly intervals with 85 mg/kg *N*-ethyl-*N*-nitrosourea (ENU, Sigma-Aldrich) in 10% ethanol in citrate buffer (pH 5.0). After identifying heritable phenotypes, affected B6 mice were out-crossed with NOD H-2^k^ congenic mice, and the F1 mice were intercrossed. DNA from affected F2 mice was scanned using simple sequence length polymorphism markers and agarose gel electrophoresis. For experiments mice were housed either under SPF or clean conventional conditions and showed the same colitis phenotype in both housing conditions.

### Assessment of Inflammation and Intestinal Permeability

Scoring of aberrant crypt architecture (score range 0–5), increased crypt length (0–3), goblet cell depletion (0–3), general leukocyte infiltration (0–3), lamina propria neutrophil counts (0–3), crypt abscesses (0–3), and epithelial damage and ulceration (0–3) was performed on the proximal and distal colon at 6, 12, and 18 wk of age (full details in Table S1). The mesenteric lymph nodes (MLNs) were dissected free of fat, disaggregated between two microscope slides, and the number of recovered leukocytes determined. 2 × 10^6^ MLN leukocytes were cultured in 1 ml of RPMI1640 containing 10% fetal calf serum and stimulated with 50 ng/ml PMA and 750 ng/ml ionomycin. The distal one-third of the colon was cleared of luminal material, weighed, washed four times in antibiotics, diced into 1 mm square pieces, and then cultured as explants for 24 h in 1 ml of RPMI1640 containing 10% fetal calf serum. MLN and explant culture supernatants were frozen at −70 °C until assayed for the inflammatory cytokines interleukin (IL)-1β, tumour necrosis factor (TNF)-α, interferon (IFN)-γ (BD Biosciences), and IL-13 (R&D Systems) according to the manufacturer's instructions. To assess intestinal permeability, mice were orally gavaged with 4 kDa FITC-dextran (Sigma, 400 mg/kg body weight in PBS), blood samples obtained at 2 and 5 h, and plasma FITC-dextran concentrations determined by measuring fluorescence at 520 nm emitted in a 96-well plate excited with a 474 nm laser using a FLA5100 scanner (Fuji) versus a FITC-dextran standard curve. Immunoglobulin coating of the faecal bacterial flora was determined using an adaptation of a flow cytometry technique used in human IBD [24].

### Induction of Colitis with Dextran Sodium Sulfate

Mice were treated with 0.5%, 2%, or 3% dextran sodium sulphate (DSS) in drinking water administered continuously for 3–63 d. Body weight, stool scores, and faecal occult blood were assessed daily. In 2% and 3% DSS experiments on day 7 blood was obtained for full blood counts and serum biochemistry, mice were humanely killed and dissected and the length of the large intestine was measured, and intestinal tissue was fixed for histological analysis. For assessment of DSS-induced inflammation, increased crypt length and goblet cell depletion were excluded from the colitis scores, because these parameters are fundamental components of the *Winnie* and *Eeyore* phenotypes. In the 0.5% DSS experiment, mice were weighed weekly, monitored daily, and humanely killed when too ill to continue based on a scoring system involving loss of body weight, diarrhoea, rectal prolapses and bleeding, behaviour, and appearance.

### Antibodies, Immunohistochemistry, Immunofluorescence, and Western Blotting

Muc2 peptides (mM2.2, CPEDRPIYDEDLKK; mM2.3, NGLKPVRVPDADNC) were synthesized (Auspep), conjugated to BSA using glutaraldehyde, emulsified in Freund's adjuvant (Invitrogen), and used to immunize rabbits. Peptide-specific antibodies were purified from rabbit serum by affinity chromatography. The 4F1 antibody reactive with the MUC2 nonglycosylated VNTR peptide repeat [25] was purified from hybridoma supernatant. We purchased antibodies against FLAG (Sigma, clone M2), GRP78 (Santa Cruz, polyclonal N-20), and β-actin (Novus Biologicals, clone AC-15). Standard immunohistochemical procedures with HRP-polymer detection were used to detect MUC2 and GRP78. Standard immunofluorescence staining for Muc2 (detected with anti-rabbit Alexa 633, Invitrogen), the lectin Dolichos biflorus agglutinin (DBA, Sigma, detected with streptavidin Alexa 488, Invitrogen), and DAPI (Invitrogen) was analysed by multitracking on a LSM510 META confocal microscope (Zeiss). Standard PAGE (NuPAGE gels, Invitrogen) and Western blotting were performed with detection by chemiluminescence or dual-label infrared fluorescence on an Odyssey instrument (Li-Cor).

### Biochemical Characterization of Muc2

Faecal matter was gently removed from the small and large intestines before the mucosa was scraped from the submucosa using a coverslip and extracted in ten volumes of 6 M guanidine HCl extraction buffer, reduced, and alkylated as described [26], and subjected to agarose gel electrophoresis and Western blotting as described [27,28].

### Cloning and Expression of Recombinant Truncated Muc2 N-Terminal Proteins

The coding sequence for the murine *Muc2* N-terminal D3 domain (AJ511872 mRNA nucleotides 2452–3696, hereafter called rMuc2-D3) was reverse transcribed and amplified from murine intestinal RNA extracted from both wild-type C57BL/6 and *Winnie* mice using Platinum *Pfx Taq* polymerase (Invitrogen) and the following primers: 5′-CCCAAGCTTCCTTGCATCCACAACAAAGA-3′, 5′-CTAGTCTAGAACCATCCTGGGTCATGTTAAG-3′. The amplicon was cloned into the HindIII and XbaI restriction sites of the pSecTag3xFLAG expression vector constructed by cloning three consecutive FLAG tags into the pSecTag vector (Invitrogen), establishing an N-terminal signal peptide followed by 3 FLAG tags, and a C-terminal myc tag. Correct cloning was confirmed by sequencing. MKN45 cells were transfected with wild-type and *Winnie* rMuc2-D3 with Lipofectamine 2000 (Invitrogen). After 48 h culture medium was replaced with serum-free medium for 24 h before collecting and concentrating it through a centrifugal membrane filter (Millipore). Cells were lysed in RIPA buffer and lysates subjected to Western blotting.

### Morphological Studies

Intestinal tissue was fixed in 10% buffered formalin or frozen in OCT (Tissue Tek) and sections were stained with hematoxylin and eosin (H&E), or with Alcian blue/Periodic Acid Schiff (PAS). For electron microscopy (EM) tissue was fixed in 4% glutaraldehyde, then post-fixed in osmium tetroxide, embedded in resin, and semi-thin sections stained with toluidine blue and thin sections with uranyl acetate.

### Gene-Expression Analysis

RNA was extracted separately from the distal and proximal large intestine of three C57BL/6, three *Winnie,* and three *Eeyore* 8-wk-old mice using Trizol (Invitrogen) and cleaned on RNeasy columns (Qiagen). RNA integrity (RNA integrity number >8) was verified using a Bio-analyser (Agilent) and equal quantities of RNA from the proximal and distal large intestine of each mouse pooled. Samples were labelled for GeneChip analysis using the One-Cycle Target Labelling and Control Reagents (Affymetrix). All steps of target labelling, hybridisation, and scanning were performed according to the manufacturer's protocol. The entire microarray dataset can be accessed at NCBI GEO Accession No. GSE9913 (http://www.ncbi.nlm.nih.gov/geo/) and a heat map of genes with altered expression is provided in Figure S1. For quantitative RT-PCR RNA was reverse-transcribed using Superscript III (Invitrogen) and amplified in a Rotor Gene RG-3000 (Corbett Life Science) using Platinum SYBR Green qPCR Supermix (Invitrogen) and the following cycling conditions and primers: *Hspa5* (95 °C 10 s; 56 °C 20 s; and 72 °C 30 s: 5′-TGCAGCAGGACATCAAGTTC-3′ and 5′-GTTTGCCCACCTCCAATATC-3′), the unspliced isoform of *XBP-1* (95 °C 10 s; 60 °C 45 s: 5′- CAGCACTCAGACTATGTGCACCTC-3′ and 5′- AAAGGATATCAGACTCAGAATCTGAAGA-3′), the spliced isoform of *Xbp-1* (95 °C 10 s; 60 °C 45 s: 5′-GAGTCCGCAGCAGGTGC-3′ and 5′-CAAAAGGATATCAGACTCAGAATCTGAA-3′) and *β-actin* (94 °C 10 s; 53 °C 30 s; 72 °C 30 s: 5′-GAAATCGTGCGTGACATCAAA-3′ and 5′-CACAGGATTCCATACCCAAGA-3′).

### Assessment of Intestinal Hypertrophy and Apoptosis

20 crypts cut in longitudinal section at each region of the intestine were measured using a micrometer. To determine the percentage of cells undergoing apoptosis, all of the epithelial cell nuclei within ten consecutive longitudinal crypt sections were counted together with the number of apoptotic bodies. Additionally, TUNEL staining was conducted with an In Situ Death Detection Kit according to the manufacturer's instructions (Roche Diagnostics) and photographed with an Olympus BX60 fluorescence microscope. To assess proliferation mice were given 100 μg/g body weight 5-bromodeoxyuridine (BrdU) i.p. 2 h prior to killing for tissue removal. Sections from paraffin-embedded Swiss rolls of the small and large intestine were antigen-retrieved by heating for 20 min in 10 mM citric acid (pH 6) and cooled to room temperature, then placed in 2 N HCl for 60 min followed by boric acid-borate buffer (pH 7.6) for 1 min and then 0.01% trypsin (in 0.5 M Tris-HCl) for 3 min. Sections were then stained with the biotinylated anti-BrdU antibody and detected with streptavidin-HRP (BD Pharmingen). BrdU-positive and -negative nuclei were counted in ten crypts from proximal and distal colon and ten crypt–villus units from the small intestine.

### Human Tissue Samples

For ultrastructural studies, intestinal tissue biopsies were obtained at colonoscopy from five female and three male patients; four of the total number had distal UC and four were unaffected individuals undergoing colorectal cancer screening. Paired biopsies from proximal and distal colon were taken for conventional H&E histology and for EM. Two of the UC patients were on no treatment, one was on maintenance thiopurine and 5-aminosalicylate treatments, and the other was on a tapering dose of prednisolone. For immunohistochemistry, archival colonic biopsy tissue was prepared from five male and five female UC patients aged 24–45 y with left-sided or extensive colitis. Three had colitis in complete endoscopic remission (one with no immunosuppressive or 5-aminosalicylate treatment), four had mild colitis (three no treatment), and three had moderately severe colitis (one no treatment). Immunohistochemical detection of the Muc2 and the ER chaperone protein GRP78 was conducted using paraffin sections from these ten UC patients and normal tissue obtained from resection margins of patients undergoing surgery for colorectal cancer. The collection of tissue and clinical data followed informed consent and was approved by the Mater Health Services Human Research Ethics Committee Approval No. 396A.

### Statistical Analyses

Due to difficulties in verifying normality of distributions when the sample size is small, we have taken a conservative approach and used the nonparametric Mann-Whitney U-test or Kruskal-Wallis test with Dunn's multiple comparison test for multiple comparisons, and data are presented using box plots. For larger datasets (*n* > 10), data were assessed with probability plots to determine if they were normally distributed prior to parametric analysis by ANOVA followed by the Bonferroni post-hoc test. Survival analysis was conducted using Kaplan-Meier plots and the Mantel log-rank test. All statistical analyses were performed using Prism v4.03 (Graphpad Software) or Systat v10.2 (Systat Software). The statistical test used and the sample sizes for individual analyses are provided within the figure legends.

## Results

### Identification of Two Novel Goblet Cell Mutants

**Figure 1:**
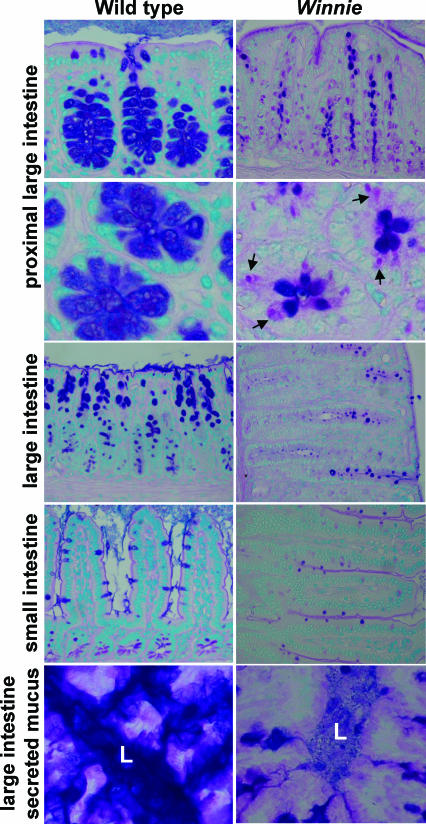
Histological Phenotype of Mice with *Muc2* Mutations PAS/Alcian blue stained intestinal sections from *Winnie* and wild-type C57BL/6 mice. Note the reduced size of Alcian blue staining thecae (stored mucin) and the presence of PAS-positive/Alcian blue negative accumulations (arrows) in *Winnie* goblet cells. L, lumen.

**Figure 2:**
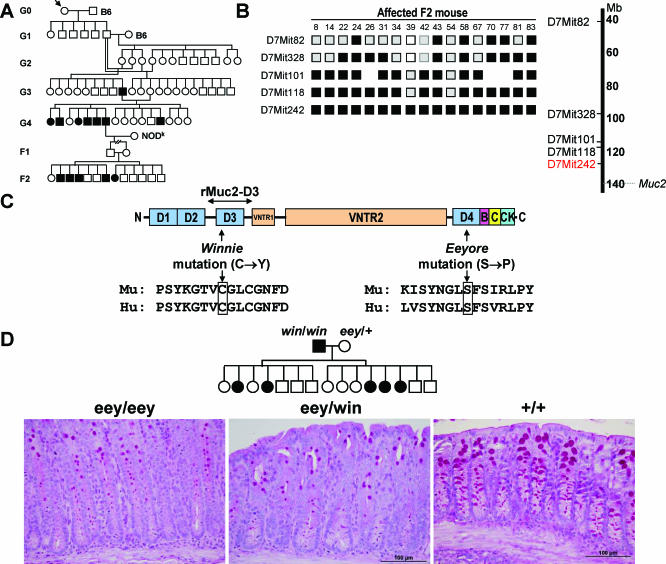
Generation and Characterization of Mice with *Muc2* Mutations (A) Dendrogram showing genesis of the *Winnie* mutation; filled symbols indicate the diarrhoea phenotype. (B) Microsatellite genotype on Chromosome 7 of 17 affected *Winnie* F2 mice. (C) Domain organization of the Muc2 protein showing the N- and C-terminal vWF D-domains (D1–D4), the C-terminal B, C, and CK domains, and the two central glycosylated tandem repeat domains (VNTR). The sites of the *Winnie* and *Eeyore* mutations are shown, together with the region cloned to express the rMuc2-D3 recombinant protein. (D) Results of complementation cross of *Win/Win* and *Eey/+*. PAS-stained sections of representative distal large intestinal histological phenotypes demonstrate noncomplementation (preservation of the *eey*/*eey* histological phenotype in *eey*/*win*).

We identified *Winnie* mice amongst the G3 progeny of an ENU-treated C57BL/6 founder by their visible phenotype of spontaneous watery diarrhoea and high incidence of rectal bleeding and prolapse. Compared with wild-type littermates, *Winnie* small and large intestines were characterized by fewer goblet cells with smaller thecae, the presence of PAS-positive Alcian blue-negative material in the cytoplasm, and a reduction in secreted mucus (Figure 1). This phenotype differs from *Muc2* ^−/−^ mice, which completely lack Alcian blue-positive mucin stored in goblet cells and secreted mucus [21]. In order to map *Winnie*, we outcrossed an affected G4 to NOD^k^ mice, and intercrossed their F1 progeny; 46/172 (27%) F2 mice had the diarrheal phenotype, consistent with a fully penetrant recessive trait (Figure 2A). Genotyping of 19 affected mice for a panel of 115 microsatellite markers mapped the mutation to a 14.5 Mb interval on Chromosome 7 encoding 198 transcripts including the *Muc2* and *Muc6* mucin genes (Figure 2B). We sequenced exons for the two mucin genes and found a single missense mutation (G9492A, GenBank accession no. AJ511872, http://www.ncbi.nlm.nih.gov) resulting in substitution of cysteine with tyrosine in the D3 domain at the N terminus of Muc2 (Figure 2C).

The possibility existed that another, unidentified mutation might be present to explain *Winnie*. We identified a second strain, *Eeyore*, from a different G0 founder, with a similar phenotype also inherited as a fully penetrant recessive trait (Figure 2D). In a *Win/Win* × *Eey/+* complementation test, eight of 22 offspring exhibited the clinical and histopathological phenotype (Figure 2D). Thus, the noncomplementation of *Winnie* with *Eeyore* provided strong evidence that both strains harbour a mutation in the same gene and that these mutations are solely responsible for the phenotype. Sequencing of *Muc2^Eey^* identified a unique missense mutation (T2996C, GenBank accession no. AJ511873) resulting in a serine to proline substitution in the C-terminal D4 domain (Figure 2C). *Winnie* and *Eeyore* remain the only characterized diarrheal/colitis phenotypes in the Australian Phenomics Facility and Phenomix Australia murine ENU mutagenesis programs.

### *Muc2* Mutant Mice Develop Spontaneous Colitis

**Figure 3:**
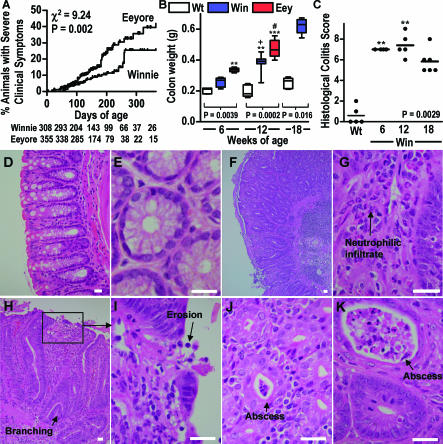
Spontaneous Colitis in Mice with Muc2 Mutations (A) Incidence of premature death or colitis-associated pathology requiring humane killing (chiefly rectal prolapse) in *Winnie* (*n* = 309) and *Eeyore* (*n* = 355) mice. Mice entering experiments or killed for other reasons were treated as censored observations (designated by upward ticks), and the number of uncensored mice remaining at 50 d intervals is included underneath the graph. Incidence rates in the two strains were compared using the Mantel Log-rank test. (B) Weight of the colon after removal of luminal faecal material in C57BL/6 (WT), *Winnie* (*Win*), and *Eeyore* (*Eey*) mice at 6 (*Eey* 6–9 wk), 12, and 18 (WT and *Win* only) wk of age, *n* = 4–9; box plots show median, quartiles, and range. (C) Histological colitis scores (see Materials and Methods) in WT and *Win* mice at 6, 12, and 18 wk of age, *n* = 4–6; scores from individual mice are shown. (D–K) Histology of normal distal colon from a C57BL/6 mouse (D and E) and examples of inflammation in the rectum (F–I) and distal large intestine (J and K) of untreated *Winnie* mice showing leukocytic infiltration (G and I), occasional branching crypts (F and H), crypt abscesses (J and K) and focal ulcerations (I); scale bars = 20 μm. Note the layer covering the mucosal surface in (F) is a granulocytic serous exudate. Statistics (B and C): *p*-values for Kruskal-Wallis nonparametric analysis are shown, Dunn's multiple comparison test versus wild type, ** *p* < 0.01, *** *p* < 0.001; versus *Win* at 6 wk, ^+^ *p* < 0.05; versus *Eey* at 6 wk, ^#^ *p* < 0.05.

The progressive incidence of rectal prolapse and colitis-associated mortality in *Winnie* and *Eeyore* mice is shown in Figure 3A. At 1 y approximately 40% of *Eeyore* and 25% of *Winnie* mice had died or were humanely killed due to the development of rectal prolapse or debility (based on a multifactorial scoring system, see Materials and Methods), whereas no wild-type littermates developed rectal prolapses. Proximal and distal colon were thickened and colon weight was greater in *Winnie* and *Eeyore* than in wild-type mice, and the thickening and weight increased progressively from 6 to 18 wk of age (Figure 3B). Despite its thickening, the colon was not shortened in mutant mice (Figure S2). Colitis was assessed histologically in *Winnie* mice at 6, 12, and 18 wk of age, revealing mild inflammation in the large intestine (Figure 3C). The inflammatory infiltrate was usually mild and did not become more severe with age. Classical signs of murine colitis, including crypt elongation, neutrophilic infiltrates, goblet cell loss, crypt abscesses, and focal epithelial erosions were present, particularly in the distal large intestine (examples shown in Figure 3F–3K).

### Extreme Susceptibility to Induced Inflammation in Mice with *Muc2* Mutations

**Figure 4:**
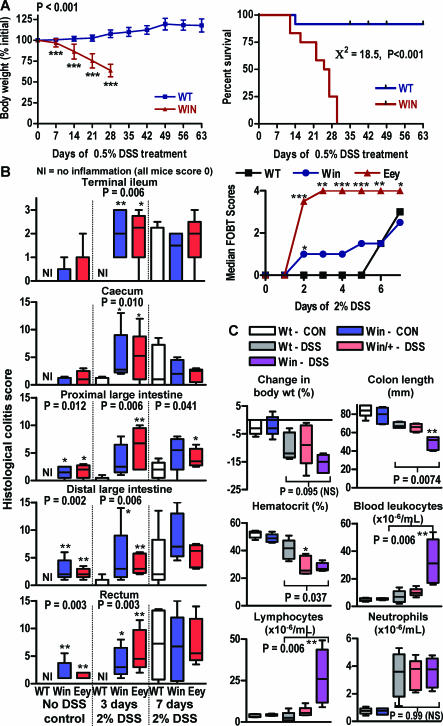
Susceptibility of Mice with *Muc2* Mutations to Dextran Sodium Sulphate–Induced Colitis (A) Wild-type (WT) C57BL/6 and *Winnie* (*Win*) mice (*n* = 12) were given 0.5% DSS in drinking water for 63 d. Body weight (mean ± standard deviation, ANOVA *p*-value shown, Bonferroni's post-hoc test versus wild-type mice, *** *p* < 0.001) and overall survival (Kaplan-Meier survival estimates and Mantel log-rank test [χ^2^-value]) are shown. (B) Wild-type C57BL/6 (WT), and *Winnie* (*Win*), and *Eeyore* (*Eey*) mice not manifesting rectal bleeding or prolapse (*n* = 6) were given 2% DSS in drinking water or water alone for 7 d. Histological colitis scores were determined on days 3 and 7 and faecal occult blood determined daily. Statistics: box plots show median, quartiles, and range; *p*-values for Kruskal-Wallis nonparametric analysis are shown, Dunn's multiple comparison test versus WT, * *p* < 0.05, ** *p* < 0.01, *** *p* < 0.001. (C) Wild-type C57BL/6, *Winnie*, and *Winnie* heterozygous (*Win*/+) mice (*n* = 5) were given 3% DSS in drinking water or drinking water alone (CON) for 7 d, at which time body weight, colon length, and haematology were assessed. Statistics: box plots show median, quartiles, and range; *p*-values for Kruskal-Wallis nonparametric analysis comparing DSS-treated WT, *Win*/+, and *Win* mice are shown, Dunn's multiple comparison test versus WT, * *p* < 0.05, ** *p* < 0.01; NS, not significant. Additional data from these experiments are shown in Figures S2 and S3.

In addition to spontaneous inflammation, *Winnie* and *Eeyore* mice exhibited increased susceptibility to environmentally induced colitis. Low doses of luminal toxins (0.5% DSS) resulted in rapid weight loss and lethal ulcerating colitis in *Winnie* by day 30, whereas wild-type mice gained weight and survived for at least 9 wk (see Figures 4A and S3). After 2% DSS, *Winnie* and *Eeyore* showed earlier faecal occult blood than did wild-type mice (after exclusion of mice with spontaneous rectal bleeding or prolapses), and higher histological colitis scores after 3 d (Figure 4B), whereas by day 7 colitis scores in wild-type mice were closer to those of mutant mice. Colitis was more pronounced in the large intestine, although some damage and inflammation were also seen in the terminal ileum (Figure S3). Although DSS is often thought to induce only colonic inflammation, damage in the ileum has been reported [29]. After oral 3% DSS for 7 d, *Winnie* mice exhibited more severe colitis than did wild-type controls, based on rate and extent of weight loss, colon length, changes in stool scores, haematocrit, blood haemoglobin, and leukocyte counts (Figures 4C and S4). All *Winnie* colons contained a massive mixed inflammatory infiltrate and almost total loss of colonic epithelium compared with much milder and intermittent colitis in wild-type mice (Figure S3).

*Winnie* heterozygotes exhibited increased rectal bleeding in response to DSS, indicating that these mutations are not simple recessive mutations under environmental stress. On day 4, only one in five wild-type mice showed rectal bleeding compared to five of five *Winnie* and five of five heterozygous mice. Mean haematocrits on day 7 were 41% for wild-type versus 28% for *Winnie* mice and 29% for heterozygotes (Figure 4C).

### Increased Intestinal Proliferation and Apoptosis in Mice with *Muc2* Mutations

**Figure 5:**
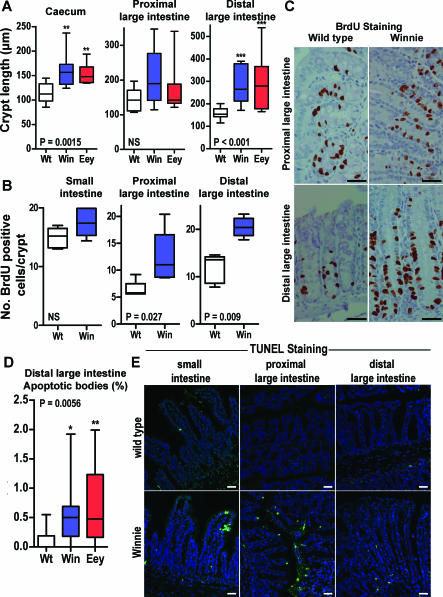
Intestinal Proliferation and Apoptosis Are Increased in Mice with *Muc2* Mutations (A) Length of caecal, proximal, and distal large intestinal crypts in wild-type C57BL/6 (WT, *n* = 12), *Winnie* (*Win*, *n* = 11), and *Eeyore* (*Eey*, *n* = 14) mice. (B) Assessment of incorporation of BrdU in the small intestine, and proximal and distal large intestine of WT (*n* = 5) and *Win* (*n* = 5) mice. For each region of the intestine in each mouse the number of BrdU-positive nuclei was counted in ten crypts (see Materials and Methods). (C) Representative examples of BrdU staining in the proximal and distal colon. (D) Increases in the percentage of apoptotic bodies in distal large intestinal crypts of the same mice as in (A). (E) TUNEL staining showing increased apoptosis in the small and large intestine of *Winnie* mice (images representative of three mice in each group). Scale bars (C and E) = 50 μm. Statistics: box plots show median, quartiles, and range; (A, B, and D) Kruskal-Wallis nonparametric analysis (*p*-values shown) with Dunn's multiple comparison test (*p < 0.05, ** *p* < 0.01, *** *p* < 0.001); (B) Mann-Whitney U-test, *p*-values shown. NS, not significant.

Both *Winnie* and *Eeyore* mice showed crypt elongation throughout the large intestine (Figure 5A) suggesting increased epithelial proliferation. This possibility was confirmed by increased BrdU incorporation in the proximal and distal colon but not in the small intestine (Figure 5B and 5C). TUNEL staining and quantitative assessment of apoptotic cells indicated that the decrease in goblet cell number (Figures 1 and 2D) in the two mutants was due to increased apoptosis in the distal colon as well as in the small intestine, caecum, and proximal and distal colon of *Winnie* mice (Figure 5D and 5E). Consistent with these morphological findings, comparison of the intestinal transcriptome (see Table S2) of *Winnie*, *Eeyore*, and wild-type mice revealed altered expression of molecules associated with regulation of epithelial growth and wound repair (increased secretory leukocyte peptidase inhibitor, epiregulin, amphiregulin, heparin-binding epithelial growth factor, lipocalin-2, stratifin, and the receptor for hyaluronan-mediated motility), cell cycle progression (increased cyclin B1 and p21), and apoptosis (increased intectin and RIP3 kinase, decreased Bcl2). In contrast to *Winnie* and *Eeyore* mice, while *Muc2* ^−/−^ mice also showed elongated crypts and increased mitosis, their rate of epithelial apoptosis was decreased compared to wild type [21]. Unlike *Muc2* ^−/−^ mice, neither *Winnie* nor *Eeyore* developed spontaneous intestinal tumours [21] nor did they show growth retardation [22].

### Altered *Muc2* Expression in Mice with *Muc2* Mutations

**Figure 6:**
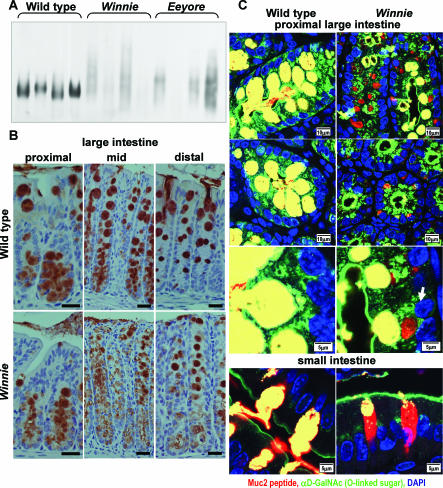
Altered Muc2 Protein Expression in Mice with *Muc2* Mutations (A) Muc2 proteins from wild-type, *Winnie*, and *Eeyore* intestinal extracts were reduced and separated by agarose gel electrophoresis and detected by Western blotting with the mM2.2 antibody. Note the reduced intensity and the altered electrophoretic migration in the mutant proteins. (B) Cellular localization of Muc2 in wild-type and *Winnie* colon tissue determined by immunohistochemistry with affinity purified mM2.2 antibody. Note the decreased size of goblet cell thecae and cytoplasmic Muc2 staining outside thecae in *Winnie*; scale bars = 25 μm. (C) Confocal microscopy of wild-type and *Winnie* goblet cells from the proximal colon and small intestine showing vacuolar accumulation of Muc2 (red) lacking O-linked sugars identified with the DBA lectin (green) in *Winnie* only. Goblet cell thecae appear yellow due to merging of the red Muc2 core peptide with the green O-linked sugar. Nuclei are stained with DAPI (blue). Scale bars as marked. Single colour images of these composites are provided in Figure S5A–S5C.

*Muc2* mRNA concentrations were decreased in the large intestine 2.3-fold in both *Winnie* and *Eeyore* compared to wild-type mice, consistent with fewer goblet cells and/or transcriptional repression of *Muc2* (see Table S3). Muc2 protein was also decreased in intestinal extracts of both *Winnie* and *Eeyore*, consistent with decreased production and/or decreased stability of secreted Muc2 (Figure 6A). Compared with the wild-type protein, migration of mutant Muc2 during agarose gel electrophoresis was more diffuse, consistent with altered glycosylation or reduction-insensitive oligomerisation.

In addition to quantitative and qualitative changes in secreted Muc2, the distribution of the mucin within the goblet cells was abnormal in *Winnie* and *Eeyore*. By immunohistochemistry (using mM2.2, a Muc2 N-terminal peptide antibody that binds independently of mucin glycosylation) Muc2 is normally confined to the thecae of goblet cells, where the mature glycosylated molecule is stored prior to secretion (Figure 6B). By contrast, in both *Winnie* and *Eeyore* mice, goblet cell thecae were smaller and fewer, and Muc2 was also present within the cytoplasm distinct from thecal structures (Figure 6B). Identical staining was seen with a C-terminal antibody (mM2.3, unpublished data). In addition, diffuse accumulations of Muc2 were seen in cells otherwise not recognizable as goblet cells because they lacked thecae, a morphological finding that has also been reported in human UC [16,30,31].

In the proximal to mid-colon there are two distinct lineages of mucus-producing cells of differing morphology [32]. Both expressed *Muc2* and exhibited the altered cellular distribution of Muc2 protein in the mutant mice, although this distribution was more pronounced in the longer-lived goblet cells at the base of the crypts. Although the entire colon and small intestine of the mutant mice harboured affected goblet cells, some areas of the colon showed greater goblet cell depletion/reduction in thecae, suggesting that the local microenvironment can exacerbate this phenotype.

Finally, we used confocal microscopy to assess mucin glycosylation using dual-label immunofluorescence labeling with the mM2.2 Muc2 antibody and the DBA lectin, which recognizes an O-linked sugar, αD-GalNAc, present on Muc2 in the small intestine and proximal to mid-colon. In wild-type proximal colon, Muc2 was confined to the thecae and was O-glycosylated (DBA lectin-positive; note that DBA lectin also reacted with some other glycoproteins in the glycocalyx and within the cytoplasm [Figure 6C]). In *Winnie* and *Eeyore* mice, where goblet cell thecae were present they contained glycosylated (DBA lectin-positive) Muc2. However, in addition, prominent cytoplasmic accumulations of non-O-glycosylated (DBA lectin-negative) Muc2 were seen within well-circumscribed vacuolar structures in virtually all goblet cells, including in some cells lacking clear goblet cell thecae (Figure 6C). These accumulations were most prominent in goblet cells in the base of the crypts in the proximal colon, but were seen in all other Muc2-producing goblet cells throughout the small and large intestine (Figure S5). Generally, accumulations were more prominent in *Eeyore* than in *Winnie* mice. Some apoptotic cells shed into the lumen contained Muc2-positive DBA lectin-negative vacuoles consistent with apoptosis of goblet cells. Notwithstanding the accumulation of Muc2 precursor, immunohistochemistry and immunofluorescence confirmed that some goblet cells were actively secreting glycosylated Muc2 in both strains of mutant mice and that Muc2 was present within luminal secretions.

### Hyperoligomerisation of Mutant Muc2 Protein

**Figure 7:**
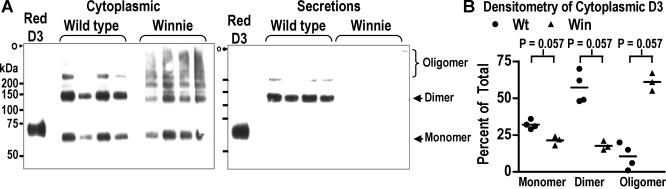
Altered Muc2 N-Terminal Oligomerisation Caused by the *Win* Mutation (A) PAGE/Western blotting analysis of oligomerisation of the rMuc2-D3 wild-type proteins and proteins with the *Win* mutation in MKN45 cells and secretions following transfection (*n* = 4 separate transfections for each group). The position of the origin (o) and markers are shown. A reduced sample (D3 Red) on each gel shows the migration of the D3 monomer. Recombinant proteins were detected with the M2 anti-FLAG antibody, and no reactivity was seen with untransfected cells. Note the hyperoligomerisation of the *Winnie* D3 domain intracellularly and its failure to be secreted. (B) Densitometric analysis of oligomerisation of the rMuc2-D3 *Winnie* and wild-type proteins in MKN-45 cellular lysates following transfection. Relative expression of monomer, dimer, and higher-order oligomers was determined by densitometry and expressed as a percentage of the total densitometric value for each sample (lane). Statistics: individual data points and *p*-values from Mann-Whitney U-tests shown.

Muc2 is secreted as an insoluble gel that is not suitable for biochemical analyses without reduction, and reduction prevents analysis of oligomerisation. Additionally, the complete *Muc2* cDNA is too large (>15 kb) to express in vitro. Therefore, to ascertain whether the *Winnie* mutation affects biosynthesis of Muc2, we cloned partial cDNAs encoding the *Winnie* and wild-type Muc2 N-terminal D3 oligomerisation domain (rMuc2-D3, Figure 2C) and expressed these as recombinant proteins in MKN45 gastric cancer cells which produce the MUC5AC mucin (but not MUC2) and are therefore likely to express mucin-specific chaperones. Western blotting of cell lysates demonstrated increased oligomerisation of the D3 proteins carrying the *Winnie* mutation (Figure 7). Wild-type D3 proteins were present intracellularly mainly as monomers and dimers, with a small amount of tetramer (11%). In contrast, 61% of *Winnie* D3 proteins were in the form of a ladder of higher-order oligomers. Wild-type D3 proteins were secreted from MKN45 cells almost exclusively as dimers with a small tetramer band, whereas, despite production intracellularly, *Winnie* D3 proteins were not secreted, thus confirming the mutation leads to a defect in biosynthesis or secretion.

### *Muc2* Mutations Lead to Accumulation in the ER

**Figure 8:**
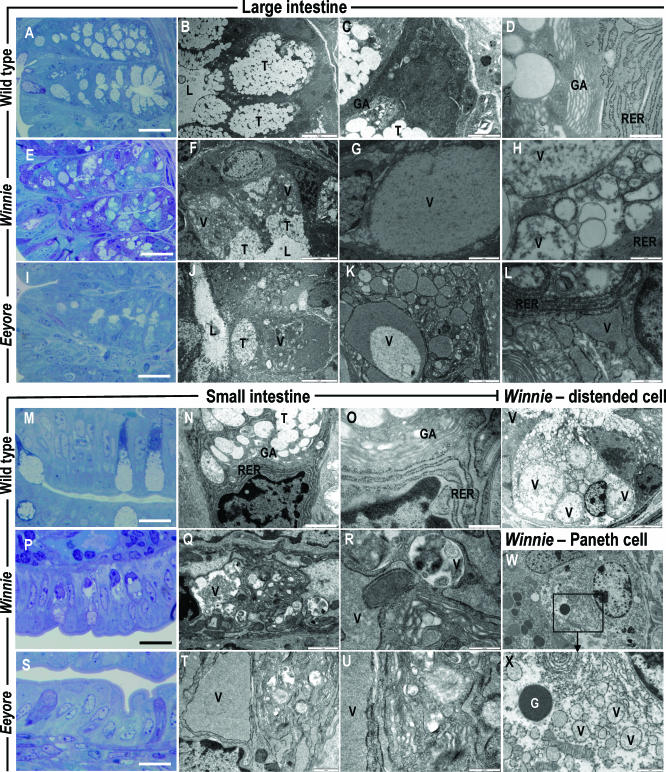
Ultrastructural Evidence of Endoplasmic Reticulum Stress in the Intestinal Epithelium of Mice with *Muc2* Mutations Semi-thin sections from resin embedded large intestine of wild-type (A), *Winnie* (E), and *Eeyore* (I) mice and small intestine of wild-type (M), *Winnie* (P), and *Eeyore* (S) mice stained with toluidine blue. Transmission electron micrographs from the large intestine (B–D, F–H, J–L, and V) and small intestine (N, O, Q, R, T, U, W, and X) of wild-type (B–D, N, and O), *Winnie* (F–H, Q, R, and V), and *Eeyore* (J–L, T, and U) mice. Note the reduced size of goblet cell thecae (indicated by a T) containing stored mucin granules and the presence of vacuoles (indicated by a V) surrounded by rough endoplasmic reticulum (RER) in *Winnie* and *Eeyore*. Other abbreviations: G, Paneth cell granule; GA, Golgi apparatus; L, lumen. Scale bars 40 μm (A, E, and I), 20 μm (L, O, and R), 5 μm (B, F, J, and U), 2 μm (C, G, K, M, P, S, and V), 1 μm (W), 50 nm (D, H, L, N, Q, and T).

Both *Muc2* mutations result in a reduction of Muc2 secretion and altered oligomerisation, but the presence of cytoplasmic accumulations of Muc2 precursor raised the possibility of additional pathogenic effects. Since alterations resulting from point mutations can result in functional and sometimes toxic effects not observed when the protein is absent, such effects could contribute to the phenotype of *Winnie* and *Eeyore* and explain differences of these mutants from *Muc2* ^−/−^ mice. To this end, we next examined both mutant strains for ultrastructural changes. Resin sections revealed that *Winnie* and *Eeyore* small and large intestines contained large numbers of cells distended with membranous vacuolar material, corresponding with the areas in which Muc2 precursor was detected by confocal microscopy. Vacuolisation was more frequent in the large intestine, and vacuolated cells usually contained stored mucin granules in thecae, demonstrating that they were goblet cells, although these thecae were smaller than in wild-type goblet cells (Figure 8A, 8E, 8I, 8M, 8P, and 8S). By transmission EM, goblet cells possessed dilated rough ER containing aggregates of variable electron density, which is the ultrastructural appearance of protein misfolding and ER stress (Figures 8F–8H, 8J–8L, 8Q, 8R, 8T, and 8U). The size and extent of these ER accumulations varied; cells with more were substantially distended, usually lacked stored mucin granules and often contained swollen mitochondria with disrupted cristae (Figure 8V). The two distinct lineages of mucus-producing cells in the proximal colon both showed vacuolisation, although vacuoles were more extensive in the longer-lived lineage that resides in the base of the crypts [32]. Some vacuoles were surrounded by membranes lacking ribosomes, possibly representing accumulations in the Golgi apparatus. Vacuole accumulation was restricted to Muc2-producing cells, with most nongoblet epithelial cells showing normal ultrastructure. However, some enterocytes and Paneth cells also contained ER vacuoles, although these vacuoles were fewer and smaller than in goblet cells, consistent with the comparatively low levels of Muc2 production that we observed in these cells by immunohistochemistry (Figure 8X).

**Figure 9:**
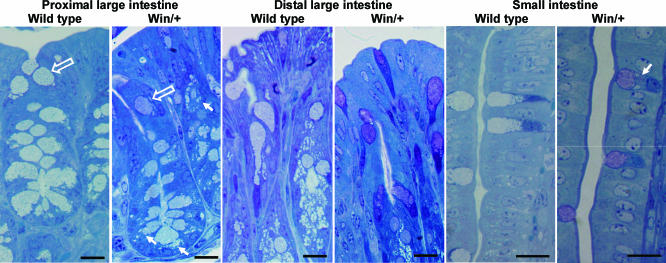
Evidence of Mucin Misfolding in Heterozygous Mice Intestinal tissue from mice heterozygous for the *Win* mutation (*Win*/+) was fixed with glutaraldehyde, embedded in resin, and semi-thin sections stained with toluidine blue. Note the variable degree of vacuolisation of the goblet cells in the base of the crypts in the proximal large intestine (solid white arrows) and the lack of/minimal vacuolisation in surface goblet cells in the proximal large intestine (hollow arrows), distal large intestine and small intestinal villi. Tissue from wild-type C57BL6 mice is shown for comparison. Scale bars = 20 μm.

Given the increased susceptibility of heterozygous mice to DSS, to assess whether one mutant allele could initiate ER stress we examined heterozygous mutants. These mice lacked the diarrhoea phenotype, and their intestinal morphology appeared normal by standard light microscopy. However, glutaraldehyde fixation and resin embedding revealed variable, though often extensive, vacuolisation in goblet cells deep in the crypts of the proximal colon (Figure 9), demonstrating subclinical pathology and establishing that these mutations are not simple mendelian recessive traits. In heterozygotes the surface goblet cell lineage in the proximal colon and the morphologically similar goblet cells in the distal colon and small intestine were mostly free of vacuolisation (Figure 9). However, vacuoles were present in some Paneth cells in the small intestine despite their lower level of Muc2 production, which may be due to the extended lifespan of these cells (unpublished data).

Accumulation of nonglycosylated Muc2 precursor (Figure 6C), altered electrophoretic migration (Figure 6A), ER vacuolisation (Figure 8), and the hyperoligomerisation of the *Winnie* D3 domain (Figure 7) together demonstrate that a proportion (but not all) of Muc2 produced in *Winnie* and *Eeyore* goblet cells is inappropriately assembled, accumulates in the ER, and leads to ER vacuolisation, smaller goblet cell thecae, and reduced secretion of mature Muc2. It appears that at some stage of their life cycle these goblet cells cease producing mature mucin, whilst still retaining Muc2 precursor in ER vacuoles (see middle right image of large intestine in Figure 6C).

### Aberrant Mucin Assembly Triggers an ER Stress Response

**Figure 10:**
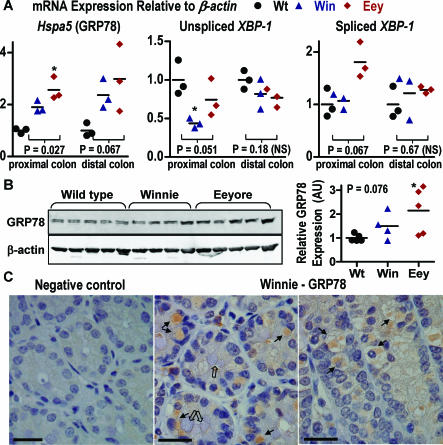
Evidence of ER Stress and UPR Activation in Mice with *Muc2* Mutations (A) mRNA expression of *hspa5* (GRP78) and the unspliced and spliced forms of *Xbp-1* in the proximal and distal colon of wild-type C57BL/6 (WT), *Winnie* (*Win*), and *Eeyore* (*Eey*) mice determined by quantitative PCR and expressed relative to β-actin with the mean ratio for the WT group in each tissue corrected to 1. Statistics: *n* = 3 individual data points shown, Kruskal-Wallis nonparametric analysis (*p*-values shown) with Dunn's multiple comparison test (*p < 0.05 versus wild type). NS, not significant. (B) Fluorescence Western blotting was used to determine the expression of GRP78 in the proximal colons of WT, *Win*, and *Eey* mice. Blots were simultaneously stained with antibodies reactive with β-actin as a loading control, and densitometry was used to quantify expression of each protein, which was then expressed as a proportion of the mean expression in wild-type mice with the mean ratio of the WT group corrected to 1. Statistics as in (A), *n* = 5. (C) Transverse crypt section (centre) and a longitudinal crypt section through the vacuole-rich base of goblet cells (right) from *Winnie* mice showing immunohistochemical detection of GRP78 in vacuolar accumulations (solid arrows) but not in thecae where present (hollow arrows). Lack of staining in the absence of the GRP78 antibody demonstrates specificity (left, negative control). Scale bars = 50 μm.

GRP78 is a heat shock protein that chaperones proteins in the ER during folding and is up-regulated in ER stress when it accumulates with misfolded proteins, ensuring they do not exit the ER normally but are removed for degradation [33]. Quantitative PCR showed a 2- to 3-fold increase in *Hspa5* (which encodes GRP78) mRNA in *Winnie* and *Eeyore* proximal and distal colon (Figure 10A). Protein misfolding engages a series of molecular events that result in reduced protein translation and transcription of genes involved in the unfolded protein response (UPR) [34]. Splicing of the *Xbp-1* mRNA by the enzyme IRE-1 is one key component of the UPR and we observed significantly decreased levels of the unspliced form of *Xbp-1* in the proximal colon of *Winnie* and increased levels of the spliced form of *Xbp-1* in the proximal colon of *Eeyore* mice (Figure 10A). The ratio of spliced to unspliced *Xbp-1*, which is used as a measure of UPR activation [35], increased 2.5- and 2.7-fold in *Winnie* and *Eeyore* proximal colon, respectively. Western blotting demonstrated increased GRP78 protein in both *Winnie* and *Eeyore* intestinal tissue (Figure 10B). In goblet cells from mutant mice GRP78 was detected immunohistochemically within the membranous accumulations, consistent with an association with misfolded Muc2, but notably not within thecae containing mucin packaged in granules for secretion (Figure 10C). Analysis of the *Winnie* and *Eeyore* intestinal transcriptome demonstrated increased transcription of the genes encoding the GRP78 and GRP94 chaperones, the γ-subunit of the SEC61 pore through which misfolded proteins exit the ER, ubiquitin peptidases, and key elements of Ca^2+^ metabolism and signalling pathways consistent with disrupted Ca^2+^ sequestration ensuing from ER stress (see Table S2).

### Immunopathology in Mice with Muc2 Mutations

**Figure 11:**
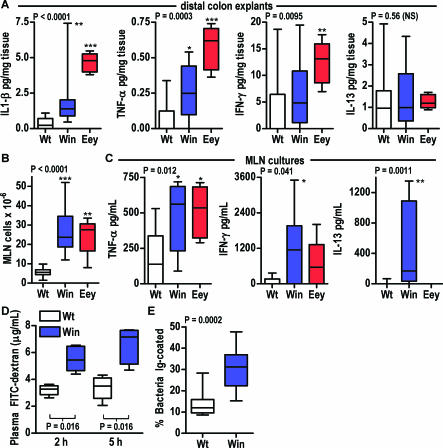
Immunopathology in Mice with *Muc2* Mutations (A) Concentrations of IL-1β, TNF-α, IFN-γ, and IL-13 in supernatants from distal colonic explants cultured for 24 h from WT (*n* = 14), *Win* (*n* = 12), and *Eey* (*n* = 5) mice at 12–18 wk of age. (B) The number of leukocytes in the MLNs was determined in C57BL/6 (WT, *n* = 16), *Winnie* (*Win*, *n* = 10), and *Eeyore* (*Eey*, *n* = 5) mice at 12 wk of age. (C) Concentrations of TNF-α, IFN-γ, and IL-13 in supernatants from 2 × 10^6^ MLN leukocytes stimulated with PMA and ionomycin and cultured for 48 h, from WT (*n* = 11), *Win* (*n* = 9), and *Eey* (*n* = 5) mice at 12–18 wk of age. For cytokine analysis samples below the sensitivity of each assay were given values of 0. (D) Plasma FITC-dextran concentrations in WT and *Win* mice (*n* = 5) 2 and 5 h following oral gavage. (E) The proportion of bacteria coated with immunoglobulin determined by flow cytometry in faecal samples from WT (*n* = 10) and *Win* (*n* = 10) mice. Statistics: box plots show median, quartiles, and range; (A and C) Kruskal-Wallis nonparametric analysis (*p*-values shown) with Dunn's multiple comparison test (*p < 0.05, ** *p* < 0.01, ***p < 0.001 versus WT); NS, not significant; (B, D, and E) Mann-Whitney U-test, *p*-values shown.

Activation of the ER stress response represents a plausible mechanism for induction of inflammation, since it is known to activate NF-κB [36,37], and a diminished mucus barrier is likely to increase exposure to prokaryotic-associated molecular patterns that activate immunity and antigens. To examine local (epithelium and lamina propria) production of cytokines, we cultured explants from the distal colon and found *Winnie* and *Eeyore* mice secreted more of the inflammatory cytokines IL-1β, TNF-α, and IFN-γ than did wild-type mice, but similar quantities of IL-13 (Figure 11A). In addition to the histological evidence of colitis, MLNs from mutant mice contained 5-fold more leukocytes than wild-type C57BL/6 mice (Figure 11B). *Winnie* and *Eeyore* MLN cells stimulated in vitro with PMA and ionomycin produced significantly more Th1 cytokines TNF-α and IFN-γ (Figure 11C). Leukocytes from *Winnie* mice also produced the Th2 cytokine IL-13, known to be produced in UC (seven of nine cultures from *Winnie* mice produced IL-13 versus low levels in just one of 11 cultures from wild-type mice; Figure 11C). IL-1β was not produced by any MLN cultures. Analysis of the intestinal transcriptome revealed molecular antimicrobial and proinflammatory changes at 8 wk of age, including: increased expression of SLPI, lipocalin-2, IL-1β, IL-1 receptor antagonist, SOCS3, guanylate nucleotide binding protein 2, CD14, immunoglobulin chains and S100 macrophage activation markers, and decreased endothelin-2 (vascular modulator of inflammation) (see Figure S6; Tables S2 and S3).

The diminished mucus barrier, stressed epithelial cells, increased epithelial apoptosis, and increased production of inflammatory cytokines all could contribute to increased intestinal permeability in *Muc2* mutant mice. Therefore we assessed permeability using a FITC-dextran absorption assay and demonstrated a 96% increase in permeability in *Winnie* mice (Figure 11D). In IBD the luminal microbial bacteria are more frequently coated with immunoglobulin [24]. Using flow cytometry we demonstrated a 2.3-fold increase in the proportion of bacteria coated with immunoglobulin in *Winnie* mice (Figure 11E).

### MUC2 Precursor Accumulation and ER Stress in Human Ulcerative Colitis

**Figure 12:**
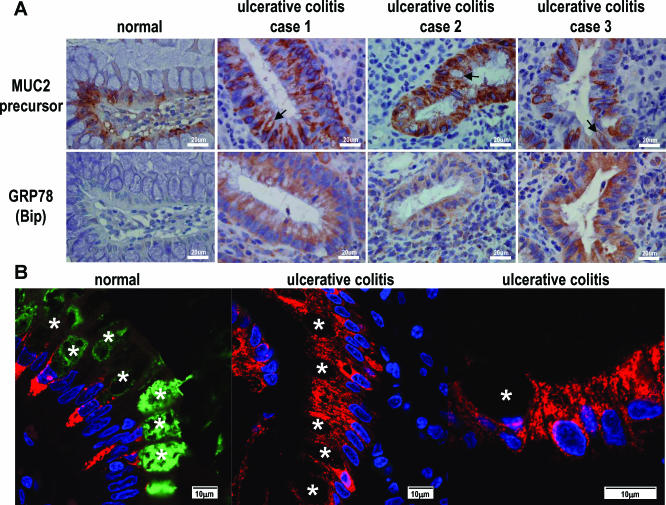
Evidence for MUC2 Precursor Accumulation and ER Stress in Human UC (A) The MUC2 precursor and GRP78 were detected immunohistochemically. One normal colonic biopsy and three representative cases of UC of ten examined are shown. Note the MUC2 precursor antibody does not react with goblet cell thecae, where present (arrows). (B) Confocal microscopy of goblet cells from the colon of an unaffected individual and two UC patients showing accumulation of the MUC2 precursor identified with antibody 4F1 (red) throughout the cytoplasm in UC goblet cells, many of which lack thecae, but not in normal colon. Note that 4F1 does not react with goblet cell thecae (identified by *) in normal or UC colon, demonstrating it cannot react with O-glycosylated MUC2 that has exited the Golgi apparatus. Note that some goblet cell thecae contain MUC2 with O-linked sugars identified with the DBA lectin (green) but that UC goblet cells are mostly DBA lectin-negative. Nuclei are stained with DAPI (blue). Single colour images of these composites are provided in Figure S7. Scale bars = 20 μm (A) and 10 μm (B).

The intracellular accumulation of membranous vacuoles observed in the *Muc2* mutant mice is reminiscent of the previously unexplained ultrastructural changes of human UC (but not Crohn's disease), which occur in both lesional and unaffected areas of intestine [38–42]. We hypothesized that these vacuoles could contain aberrantly assembled MUC2. Immunohistochemical detection of the MUC2 precursor with the 4F1 antibody in human UC intestines demonstrated a pattern of staining consistent with accumulation of misfolded MUC2 in eight of ten patients (examples in Figure 12A). The MUC2 precursor was present throughout the cytoplasm of goblet cells, except in the theca (see arrows). Additionally, many cells lacking goblet cell morphology stained intensely, as described in previous studies with similar antibodies against the peptide core of the MUC2 O-glycosylated domain [16,30,31]. This observation contrasts with that in normal human intestinal goblet cells, in which staining was restricted to the perinuclear region where the ER is located. Alterations in mucin oligosaccharides are characteristic of UC [15,43], and in this light, previous studies of MUC2 precursor antibody staining have interpreted the increased staining as being a consequence of reduced size or density of O-linked oligosaccharides. However, using confocal microscopy we show that, both in normal colon and in UC colon, the 4F1 antibody does not react with either glycosylated mucin or secreted mucus (Figures 12B and S7), proving that the 4F1 staining represents accumulation of non-O-glycosylated precursor in UC. In normal colon 4F1 reactivity is exclusively with the basal perinuclear, mostly subnuclear, region of the goblet cell, whereas in UC 4F1 reactivity was often seen dispersed throughout the entire cytoplasm as would be predicted during ER stress. All UC samples with accumulation of the MUC2 precursor showed concomitant expression of GRP78, consistent with protein misfolding and ER stress, whereas GRP78 was not detected immunohistochemically in normal intestine (Figure 12A).

Using EM we found that intestinal goblet cell morphology in four UC patients with mild distal colitis showed similarities to that in *Winnie* and *Eeyore* mice, even in noninflamed areas of the colon, whereas four noninflamed controls showed no such changes (examples shown in Figure S8), as has been described previously [38–42]. Goblet cell density was reduced in UC, thecae were typically smaller, and vacuolar accumulations were present. EM showed dilated rough ER and often very large vacuoles surrounded by membranes lacking ribosomes, suggestive of accumulations in the Golgi apparatus, confirming the previous studies. Whilst non-UC intestinal goblet cell thecae contained densely packed mucin granules, UC goblet cell thecae often contained diffuse material of differing electron density dispersed between granules. The variable appearance of the granules in the UC thecae, which was not seen in *Winnie* and *Eeyore*, is consistent with either inappropriate mucin packaging in the Golgi or a greater proportion of nonmucin goblet cell secretions.

## Discussion

This study demonstrates that the intestinal secreted mucin is prone to misfolding/aberrant assembly and that misfolding causes substantial ER stress, morphological goblet cell pathology, premature goblet cell apoptosis, and the development of chronic intestinal inflammation. We have described two strains of mice with ENU-induced allelic variants of *Muc2* and phenotypes that closely model human colitis. To our knowledge *Winnie* and *Eeyore* are the first animal models of spontaneous intestinal inflammation arising due to single missense mutations in any gene. Their phenotype is characterized by diarrhoea, decreased goblet cell number, smaller goblet cell thecae, accumulation of Muc2 precursor in ER vacuoles within the goblet cell cytoplasm, increased epithelial cell proliferation and apoptosis, depletion of the secreted mucus layer, and spontaneous inflammation. The resulting pattern of inflammation with intestinal production of IL-1β and TNF-α, and the production of both Th1 (TNF-α and IFN-γ) and Th2 (IL-13) cytokines by MLN leukocytes, resembles the complex and somewhat inconsistent patterns of cytokine production described in UC [44–52]. The IL-13 could be sourced from natural killer T cells, which have been shown to produce IL-13 in UC [50], and in oxazolone-induced colitis in mice, which is considered a UC-like model [53], or from natural killer cells, which are known to produce IL-13 in the intestine during some infections [54]. The increased intestinal permeability and increased coating of luminal bacteria with immunoglobulin also mirror the IBD phenotype [24,55].

The *Winnie* and *Eeyore* mutants provide new insights into the complex relationship between goblet cell physiology, mucin production, and intestinal inflammation. Several findings suggest that the phenotype is not accounted for simply by a reduction in the mucus layer. Both *Win* and *Eey* alleles cause biosynthetic defects leading to accumulation of Muc2 precursor, ER vacuolisation and stress, and activation of the UPR. Furthermore, despite an apparently normal mucus barrier, heterozygotes exhibited a restricted intestinal ER stress phenotype and an intermediate phenotype of sensitivity to luminal toxins, consistent with ER stress contributing to epithelial damage and environmentally induced inflammation. Finally, the presence of a large number of stressed cells within the intestinal epithelium and the production of IL-1β, TNF-α, and IL-13, are likely to attenuate epithelial tight junction integrity, leading to the impaired epithelial barrier function we demonstrate [52,56]. Direct biochemical links between ER stress and both local and systemic inflammation suggest that ER stress directly promotes inflammation [57,58], including inducing activation of NF-κB via multiple pathways within cells experiencing ER stress [36,37,59,60]. NF-κB activation is considered central to intestinal inflammation [61,62] and has been observed in unaffected tissues from patients with UC and in asymptomatic identical twins [63]. During intestinal ER stress it is likely that NF-κB activation occurs in epithelial cells both as a direct result of ER stress and due to increased exposure to microbial Toll-like receptor ligands as a result of a diminished mucus barrier and increased epithelial permeability.

While ER stress provides one contributory pathway to intestinal pathology, it is inherently coupled with a reduction in quantity and quality of the mucus layer. The mutant strains contain fewer goblet cells due to apoptosis, less Muc2 is likely to be secreted per goblet cell due to misfolding and the UPR-initiated translational block, and the mucin that is secreted could be incorrectly oligomerised and affect the rheological properties of mucus. The relative importance of ER stress and changes to the mucus barrier cannot be easily distinguished, because they are inextricably linked and may well act in concert. However, several lines of evidence suggest that Muc2 depletion in the absence of ER stress is not sufficient to cause colitis. First, even in *Muc2* ^−/−^ mice the colitis phenotype appears to arise only on a permissive genetic background [21,22]. Second, specific depletion of approximately 60% of goblet cells in mITF/DT-A transgenic mice did not trigger spontaneous inflammation and reduced susceptibility to DSS-induced colonic injury [23]. Third, *Gfi1* ^−/−^ mice, which produce very few intestinal secretory lineage cells, including goblet cells, do not show intestinal inflammation [64]. There are substantial phenotypic differences between the *Muc2* mutants and *Muc2* ^−/−^ mice. Although *Muc2* ^−/−^ mice lack goblet cell thecae, they maintain normal goblet cell numbers and production of other secretions [21]. *Muc2* ^−/−^ show neo-expression in the colon of the Muc6 gastric mucin [22], whereas, similar to UC [65], Muc6 was not expressed by *Winnie* and *Eeyore*. Muc2 deficiency causes increased mitosis/crypt hyperproliferation [21] and, on a 129/Sv background but not a C57BL/6J × 129/Sv background, growth retardation, diarrhoea, occasional rectal prolapses, focal erosions, and mild inflammation in the distal colon and increased susceptibility to DSS-induced colitis [22]. However, an increase in epithelial apoptosis occurs in *Winnie* and *Eeyore* mice and in humans with UC, whereas in contrast complete loss of Muc2 reduces apoptosis [21]. Even if one argues that the dominant feature causing colitis in *Winnie* and *Eeyore* is the depletion of the mucus barrier, then these two spontaneously occurring models demonstrate how mucin depletion sufficient to initiate colitis can occur via simple missense mutations in *Muc2*, and that heterozygosity for such mutations is sufficient to cause enhanced susceptibility to environmental insults.

Previous evidence also supports a role for ER stress in intestinal inflammation. Mice lacking IRE1β, a key initiator of the UPR, are more susceptible to DSS-induced colitis [66]. Furthermore, ER stress proteins are up-regulated in DSS-induced colitis in mice and in UC in humans [57], and GRP78 expression is up-regulated in ex vivo-cultured intestinal epithelial cells from patients with colitis [59]. Ultrastructural studies in UC and Crohn's disease [38–42,67], together with data reported here of both inflamed and noninflamed UC colon, report cellular changes in UC but not Crohn's disease consistent with protein misfolding and ER and/or Golgi apparatus stress, similar to those seen in *Winnie* and *Eeyore* mice. In addition we report ultrastructural changes in goblet cell thecae in UC suggestive of either inappropriate granule formation or premature dissolution of stored granules prior to secretion. Importantly, the presence of aberrant goblet cell morphology in unaffected proximal colon of UC patients, as reported here and previously [38], suggests that this defect precedes rather than follows inflammation. Furthermore, previous immunohistochemical studies with VNTR peptide core-reactive MUC2 antibodies [16,30,31] reported diffuse staining in UC goblet cells and, to a lesser extent, in colonic Crohn's disease, that is independent of the degree of inflammation [16]. We have replicated this finding and demonstrate that*,* rather than this staining pattern reflecting glycosylation changes in mature MUC2, it is the precursor that accumulates in a similar pattern to that seen in *Winnie* and *Eeyore*. We also show concomitant expression of GRP78, providing biochemical evidence of ER stress.

The size and complexity of Muc2 are likely to render it particularly susceptible to aberrations in folding and assembly of the oligomeric complex. Hyperoligomerisation of the recombinant *Winnie* D3 domain caused retention in the secretory pathway, which is consistent with altered Muc2 homo-oligomerisation being the cause of ER retention in vivo. Even though N-terminal oligomerisation of Muc2 is thought to occur in the Golgi, the presence of ribosomes in vacuole membranes and the lack of O-glycans on the accumulated precursor show that aberrant oligomerisation with the *Win* mutation occurs in the ER. The complex nature of misfolded oligomerised Muc2 is likely to present a major challenge to the ER machinery responsible for denaturing and removing misfolded proteins from the ER for degradation [33,68]. The more severe ER stress in the goblet cells deep in the crypts of the proximal large intestine is consistent with the longer lifespan (15–21 d) of these cells compared with the surface goblet cells in the proximal colon and goblet cells in the distal colon and small intestine (4–5 d) [32]. Thus the extended lifespan of goblet cells following mucosal damage could unmask genetic susceptibilities to ER stress and amplify the effects of environmental ER stressors.

Based on evidence from *Winnie* and *Eeyore*, single nucleotide polymorphisms (SNPs) in oligomerisation domain-encoding exons of *MUC2* may identify individuals predisposed to intestinal disease. The D-domains of MUC2 share very high homology with the blood clotting glycoprotein von Willebrand Factor (vWF) which also forms homo-oligomers. Autosomal dominant and recessive mutations in all four D-domains of vWF cause von Willebrand disease [69], typically via altered homo-oligomerisation, and are therefore analogous to the *Win* and *Eey Muc2* mutations. A single genome-wide scan has identified an IBD susceptibility locus at Chromosome 11p, which contains *MUC2* within a cluster of four mucin genes at 11p15.5 [70]. A study of *MUC2* polymorphisms found no association with UC, but examined only tandem repeat length polymorphisms incapable of distinguishing underlying SNPs [71]. Interestingly, a study examining a single MUC2 D1 oligomerisation domain polymorphism found an association with Crohn's disease but not UC [72]. There are at least 30 nonsynonomous SNPs in the exons encoding MUC2 oligomerisation domains, warranting comprehensive studies in IBD focused on these *MUC2* exons.

Although MUC2 is the most likely candidate for misfolding mutations, mutations in genes encoding any proteins with a predilection for misfolding, as well as in the genes encoding ER chaperones, elements of the UPR, and/or detoxification proteins (e.g., *MDR1*) could trigger the ER stress pathway to colitis. The resultant UPR could lead to a translational block decreasing MUC2 synthesis, and the ER stress could cause MUC2 misfolding, even in the absence of mutations in MUC2, duplicating the ER stress/reduced mucus barrier phenotype. Similarly, inappropriate responses to the normal level of misfolding occurring in secretory cells could trigger the ER stress pathway to intestinal inflammation. Highlighting likely interindividual differences in the response to misfolded proteins, it was recently shown that the class of genes with the highest level of inherited variation in expression were genes involved in the UPR [73].

Thus, a complex genetic basis for UC but involving the same pathway revealed by single alleles with strong effects (such as *Win* and *Eey*) is plausible. Furthermore, this mechanism could account for a complex interaction with environmental triggers. Candidate triggers include intestinal viruses, bacteria, dietary toxins, reactive oxygen species, and bile salts, which can directly cause or exacerbate ER stress. Furthermore, local inflammation itself can also enhance ER stress via the actions of reactive oxygen species and TNF-α [74], demonstrating how a chronic cycle of ER stress, mucus depletion, and inflammation could arise. Conversely, some local factors may ameliorate the consequences of ER stress; for example, IL-10 has recently been shown to modulate the UPR in intestinal cells by inhibiting nuclear translocation of the ATF6 transcription factor [59]. Changes in production of other goblet cell molecules involved in barrier function and/or activation of immunity during goblet cell ER stress could also have important impacts. For example, the gene encoding RELM-β which has been reported to have both pro- and anti-inflammatory activities [75,76], shows altered expression in *Winnie* and *Eeyore* (Table S3). Thus the severity of ER stress, the nature of the UPR, and the degree of inflammation in response to intestinal ER stress are likely to be dependent on a range of other genetic factors, consistent with the multiallelic nature of IBD.

### Limitations of This Study

Although the study shows that mice with aberrant mucin assembly and ER stress develop inflammation similar to human UC, the cellular and molecular pathways leading to inflammation remain to be fully characterized. There are also differences in the physiology of the intestine in the mouse, including the distribution and lifespan of goblet cells, that could influence translation of our findings to human physiology. Our study confirms previous studies showing signs of ER stress in UC but does not ascertain the genetic or environmental drivers of ER stress in UC, relate the ER stress to differing clinical phenotypes, nor explore the effects of treatment. Comprehensive, appropriately powered studies combining genetics, clinical pathology (including biochemical and ultrastructural assessment of ER stress), and environmental epidemiology are required to address the involvement of ER stress in human IBD.

### Conclusions

**Figure 13:**
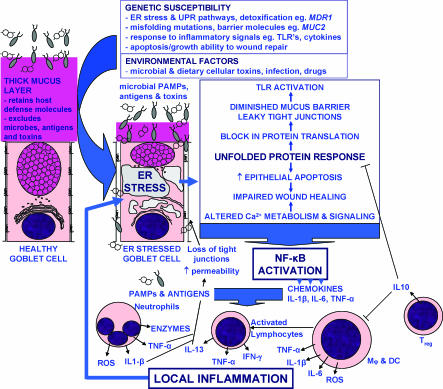
A Model for ER Stress in the Pathophysiology of Intestinal Inflammation This model recognizes that genetic and environmental factors can combine to invoke ER stress, reduced mucus production, and inflammation. Local inflammation can then exacerbate ER stress, potentially establishing a chronic cycle of inflammation in susceptible individuals. Abbreviations: ER, endoplasmic reticulum; Mϕ, macrophage; MDR1, multi-drug resistance gene 1; PAMP, prokaryocyte-associated molecular pattern; ROS, reactive oxygen species; TLR, toll-like receptor; UPR, unfolded protein response.

The evidence is consistent with a model of UC pathogenesis (Figure 13) in which ER stress due to genetic susceptibility and/or environmental factors results in MUC2 precursor accumulation and decreased production of glycosylated MUC2 by individual goblet cells leading to smaller/loss of goblet cell thecae, a reduced lifespan of individual goblet cells, and a decreased total MUC2 production. In this scenario a diminished mucus barrier would lead to greater toxin and antigen exposure, driving further ER stress, with both the depleted mucus barrier and ER stress triggering local mucosal inflammation. Local inflammatory cytokines could increase epithelial permeability and directly exacerbate ER stress through reactive oxygen species production, leading to an unresolving cycle of epithelial damage and inflammation. In this model of colitis, goblet cell ER stress and a diminished mucus barrier are intrinsically linked concurrent events, irrespective of whether the trigger is a genetic lesion predisposing to misfolding, a lesion resulting in an inappropriate UPR, or an environmental insult.

## Supporting Information

Figure S1 Heat Map Showing Relative Expression of Genes Altered in both *Muc2* Mutant Mouse Strains Hierarchical clustering of 414 probe sets that were considered up-regulated or down-regulated in intestinal RNA in at least 2/3 *Eeyore* and 2/3 *Winnie* mice compared to expression in at least 2/3 C57BL/6 wild-type mice. Heat map was generated using dChip2006, with each probe set represented as normalized signal minus mean divided by standard deviation using Euclidean distance metric and average linkage method. Row names are concatenated Affymetrix Mouse 430_2 probe ID and the Unigene symbol. The entire microarray data set can be accessed at NCBI GEO accession no. GSE9913 (http://www.ncbi.nlm.nih.gov/geo/).
(312 KB PDF)

Figure S2 Colon Length in Wild-Type and *Winnie* Mice at 6 and 12 Weeks of Age Statistics: box plots show median, quartiles, and range; *n* = 5–6; no statistical difference by Kruskal-Wallis nonparametric analysis.
(100 KB PDF)

Figure S3 Susceptibility of *Winnie* and *Eeyore* Mice to Dextran Sodium Sulphate–Induced Colitis—Histological Examples of Colitis (A) Wild-type C57BL/6 (WT), *Winnie* (*Win*), and *Eeyore* (*Eey*) mice were given 2% DSS in drinking water or water alone for 7 d. Representative examples of histology showing colitis on days 3 and 7, and small intestinal pathology on day 7, are shown. Scale bars = 100 μm.
(B) Wild-type C57BL/6 and *Winnie* mice were given 3% DSS in drinking water or drinking water alone (CON) for 7 d; representative examples of histological colitis are shown. Scale bars = 300 μm.
(C) Representative examples of ulcerating colitis in *Winnie* mice given 0.5% DSS for 4 wk.
(2.6 MB PDF)

Figure S4 Susceptibility of *Winnie* and *Eeyore* Mice to Dextran Sodium Sulphate–Induced Colitis—Additional Clinical, Biochemical, and Haematological Data Wild-type C57BL/6, *Winnie,* and *Winnie* heterozygous (*Win*/+) mice were given 3% DSS in drinking water or drinking water alone (CON) for 7 d, at which time serum amyloid protein, serum triglycerides, and blood monocyte levels and haemoglobin concentrations were assessed. Rectal bleeding and stool scores were assessed daily. Statistics: box plots show median, quartiles, and range; *n* = 5; *p*-values for Kruskal-Wallis nonparametric analysis comparing DSS-treated WT, *Win*/+, and *Win* mice are shown, Dunn's multiple comparison test versus WT *p < 0.05, **p < 0.01.
(117 KB PDF)

Figure S5 Demonstration of Muc2 Precursor Accumulation in *Muc2* Mutant Mice by Confocal Microscopy (A–C) Individual colour confocal images of the composite confocal images shown in Figure 6C.
(D) Staining of proximal colon in an *Eeyore* mouse.
(E) Negative control sections; note no background staining is seen with omission of the Muc2 antibody or DBA lectin (negative control).
Staining as indicated, scale bars = 10 μm.
(4.6 MB PDF)

Figure S6 Relative Expression of Genes Classified by Ontology into Those Encoding Cytokines and Cytokine Receptors, Chemokines and Chemokine Receptors, Interferons and Interferon-Inducible Proteins, and Leukocyte-Signalling Proteins in *Winnie* Compared to C57BL/6 Wild-Type Mice The signal intensity for each related probe_ID was averaged within the *Winnie* and wild-type mice cohorts (*n* = 3) and graphed on a log10 scale. The grey line represents the 1:1 signal intensity intersection. Each spot has been coded: red for >2 *Winnie*:wild type, green for <0.5 *Winnie*:wild type, and grey for all values >0.5 and <2.0. The entire microarray dataset can be accessed at NCBI GEO Accession No. GSE9913 (http://www.ncbi.nlm.nih.gov/geo/).
(57 KB PDF)

Figure S7 Evidence of MUC2 Precursor Accumulation in Ulcerative Colitis Individual confocal images of the composite confocal images shown in Figure 12B. Staining with the MUC2 precursor antibody 4F1, DBA lectin, and DAPI as indicated. Scale bars = 10 μm.
(560 KB PDF)

Figure S8 Morphological Evidence of ER Stress in Ulcerative Colitis Intestinal biopsies from two healthy individuals, the unaffected proximal colon of three UC patients, and the affected distal colon of one of those UC patients, were examined by light (left) and electron (right) microscopy. Arrows indicate the presence of vacuoles in goblet cells. Abbreviations: NG, nongranular material; RER, rough endoplasmic reticulum; T, theca/mucin granules; V, vacuole. Scale bars are individually annotated.
(2.9 MB PDF)

Table S1 Histological Scoring of Murine Colitis (67 KB DOC)

Table S2 Comparison of the Intestinal Transcriptome of C57BL/6, *Winnie* and *Eeyore* Mice—Genes Involved in ER Stress, Antimicrobial Defence, Wound Repair, Epithelial Growth, Cell Cycle, and Apoptosis (61 KB DOC)

Table S3 Comparison of the Intestinal Transcriptome of C57BL/6, *Winnie* and *Eeyore* Mice—Genes Involved in Inflammation, Metabolism, Detoxification, and the Mucus Barrier (60 KB DOC)

## Abbreviations

DBA: *Dolichos biflorus* agglutinin
DSS: dextran sodium sulphate
EM: electron microscopy
ENU: *N*-ethyl-*N*-nitrosourea
IBD: inflammatory bowel disease(s)
MLN: mesenteric lymph node
SNP: single nucleotide polymorphism
UC: ulcerative colitis
UPR: unfolded protein response
vWF: von Willebrand factor
WT: wild type
