# Supplementary material for: Aberrant Mucin Assembly in Mice Causes Endoplasmic Reticulum Stress and Spontaneous Inflammation Resembling Ulcerative Colitis
Source: PLoS Med. 2008 Mar 4;5(3):e54. doi: 10.1371/journal.pmed.0050054 (PMC2270292; doi:10.1371/journal.pmed.0050054)
Supplement: Supplementary file 3 [file pmed.0050054.sg003.pdf]

Figure S3

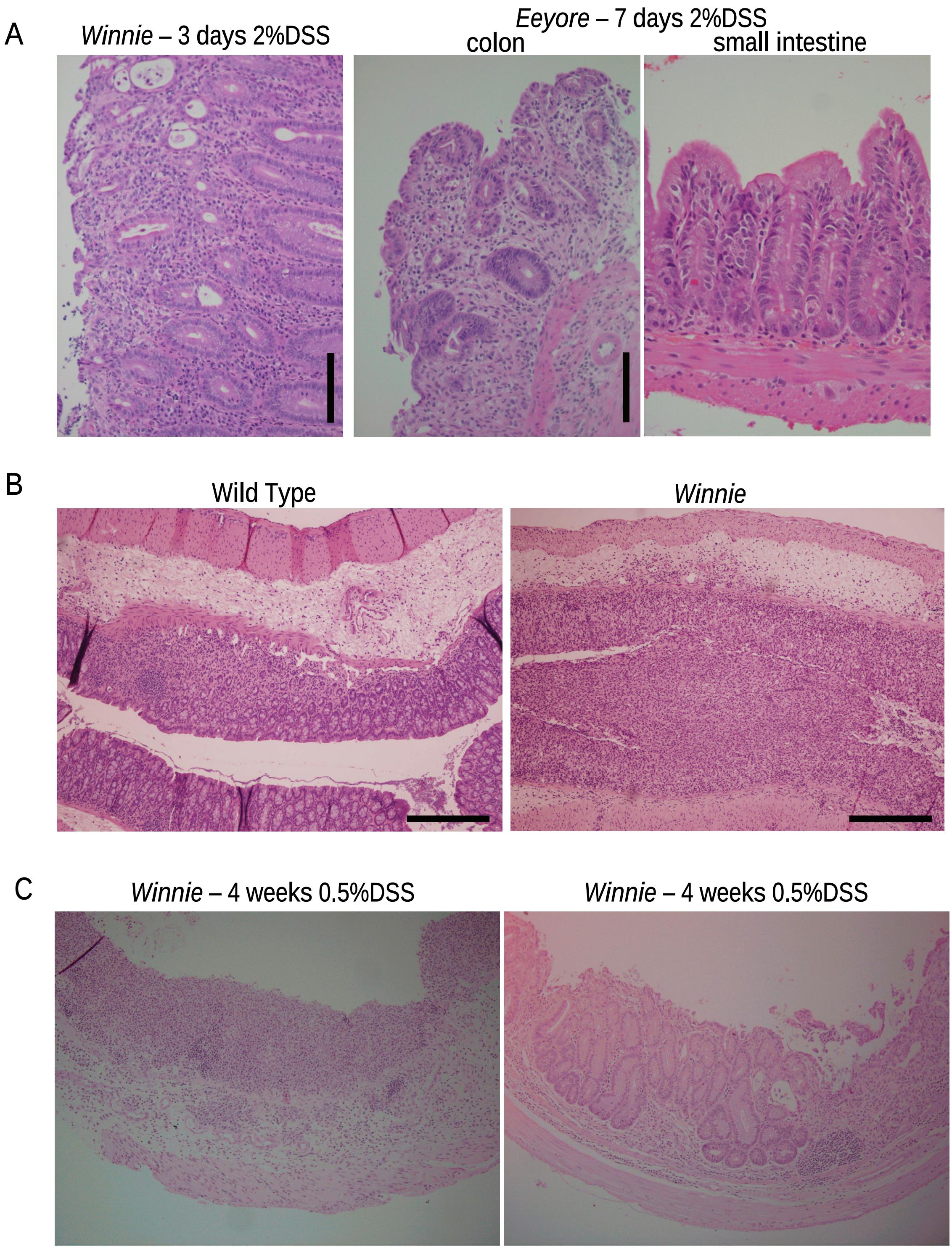
A
Winnie – 3 days 2%DSS
Eeyore – 7 days 2%DSS
colon
small intestine
B
Wild Type
Winnie
C
Winnie – 4 weeks 0.5%DSS
Winnie – 4 weeks 0.5%DSS
