# Supplementary material for: Aberrant Mucin Assembly in Mice Causes Endoplasmic Reticulum Stress and Spontaneous Inflammation Resembling Ulcerative Colitis
Source: PLoS Med. 2008 Mar 4;5(3):e54. doi: 10.1371/journal.pmed.0050054 (PMC2270292; doi:10.1371/journal.pmed.0050054)
Supplement: Supplementary file 4 [file pmed.0050054.sg004.pdf]

Figure S4

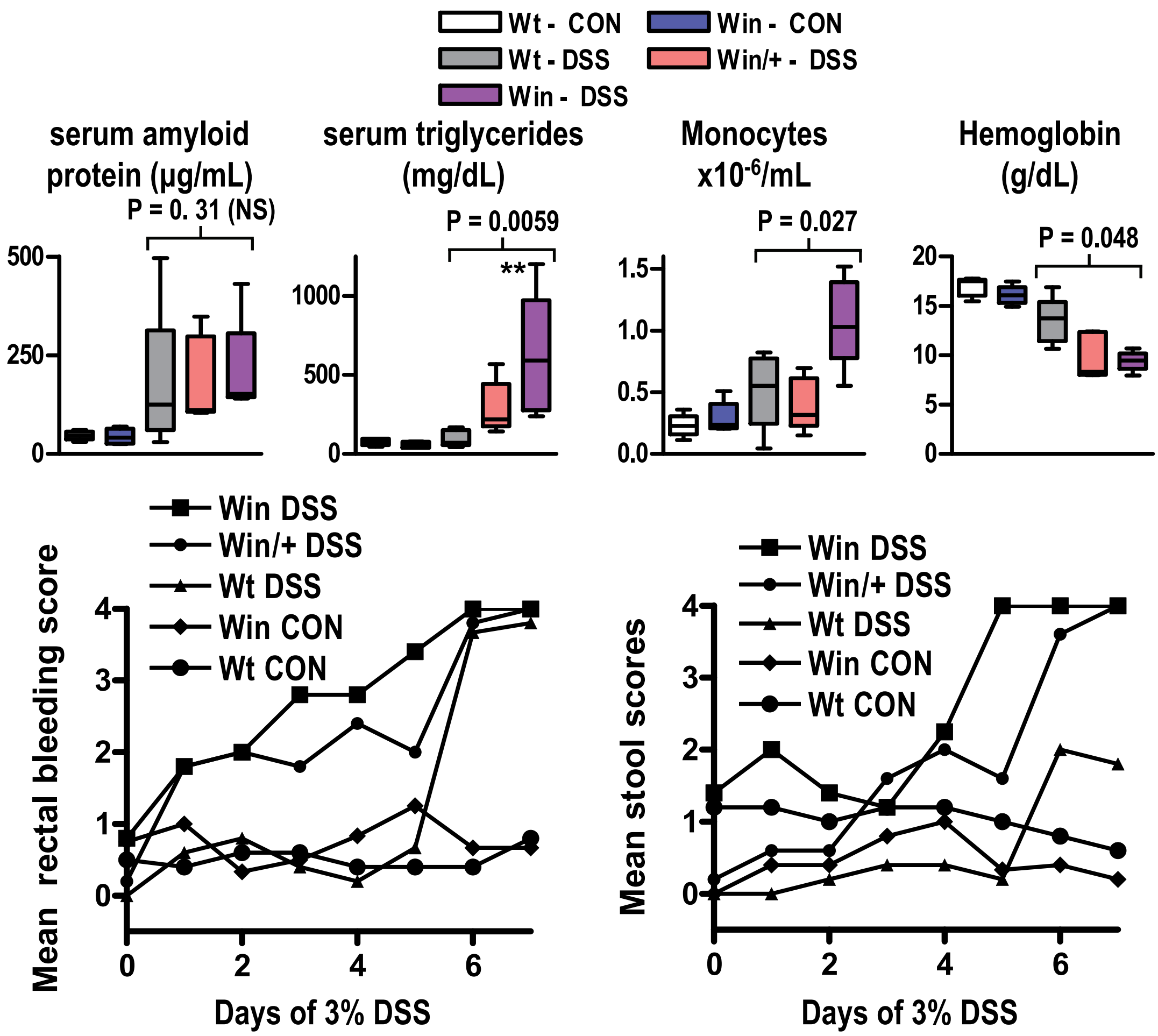

Wt - CON
Win - CON
Wt - DSS
Win/+ - DSS
Win - DSS
serum amyloid protein (µg/mL)
P = 0. 31 (NS)
500
250
0
serum triglycerides (mg/dL)
P = 0.0059
**
1000
500
0
Monocytes x10-6/mL
P = 0.027
1.5
1.0
0.5
0.0
Hemoglobin (g/dL)
P = 0.048
20
15
10
5
0
Win DSS
Win/+ DSS
Wt DSS
Win CON
Wt CON
Mean rectal bleeding score
Days of 3% DSS
Mean stool scores
Days of 3% DSS
