# Supplementary material for: Aberrant Mucin Assembly in Mice Causes Endoplasmic Reticulum Stress and Spontaneous Inflammation Resembling Ulcerative Colitis
Source: PLoS Med. 2008 Mar 4;5(3):e54. doi: 10.1371/journal.pmed.0050054 (PMC2270292; doi:10.1371/journal.pmed.0050054)
Supplement: Supplementary file 5 [file pmed.0050054.sg005.pdf]

Figure S5

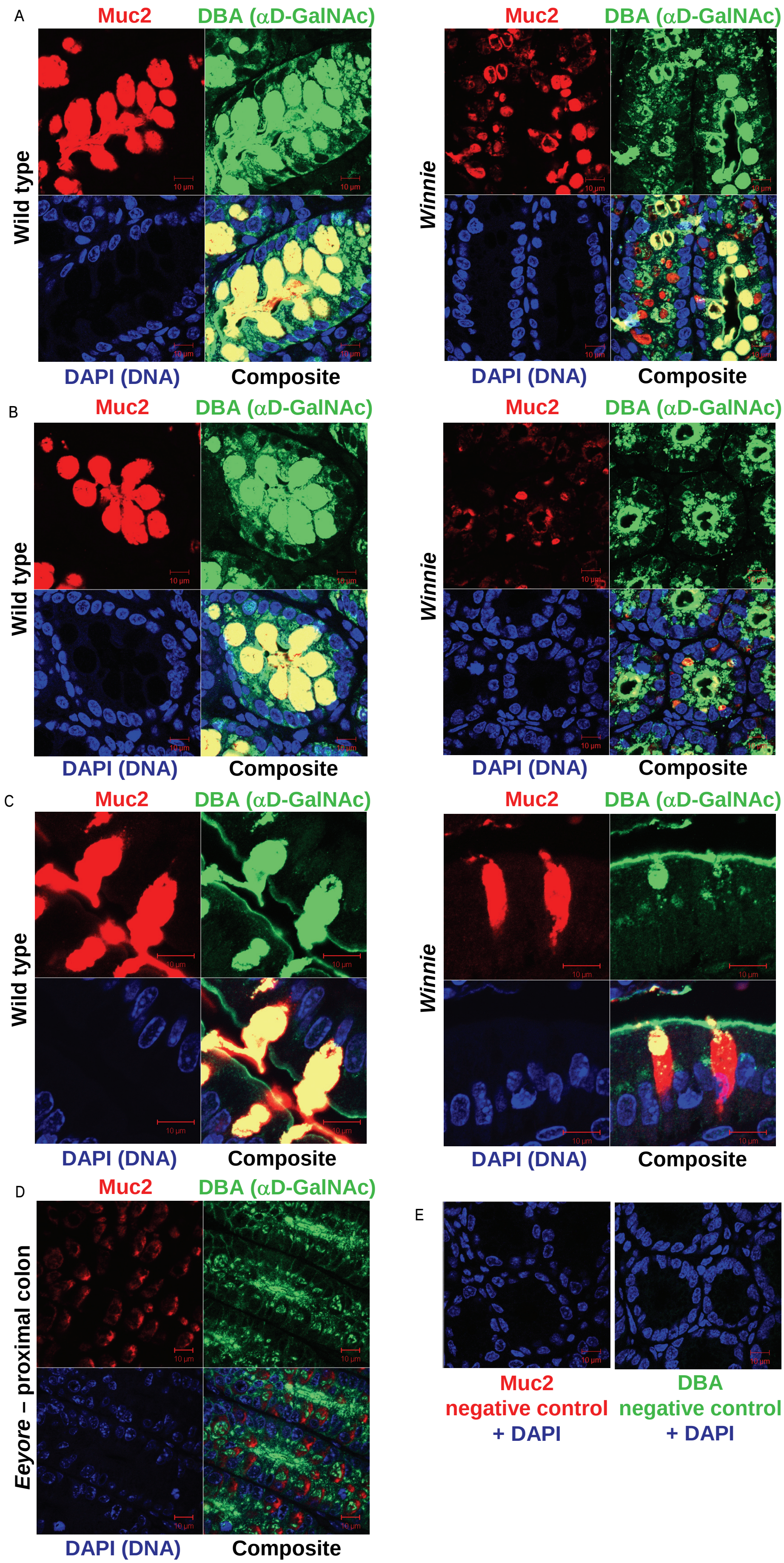
A
Muc2
DBA (αD-GalNAc)
Wild type
DAPI (DNA)
Composite
Muc2
DBA (αD-GalNAc)
Winnie
DAPI (DNA)
Composite
B
Muc2
DBA (αD-GalNAc)
Wild type
DAPI (DNA)
Composite
Muc2
DBA (αD-GalNAc)
Winnie
DAPI (DNA)
Composite
C
Muc2
DBA (αD-GalNAc)
Wild type
DAPI (DNA)
Composite
Muc2
DBA (αD-GalNAc)
Winnie
DAPI (DNA)
Composite
D
Muc2
DBA (αD-GalNAc)
Eeyore – proximal colon
DAPI (DNA)
Composite
E
Muc2
negative control
+ DAPI
DBA
negative control
+ DAPI
