# Supplementary material for: Aberrant Mucin Assembly in Mice Causes Endoplasmic Reticulum Stress and Spontaneous Inflammation Resembling Ulcerative Colitis
Source: PLoS Med. 2008 Mar 4;5(3):e54. doi: 10.1371/journal.pmed.0050054 (PMC2270292; doi:10.1371/journal.pmed.0050054)
Supplement: Supplementary file 6 [file pmed.0050054.sg006.pdf]

Figure S6

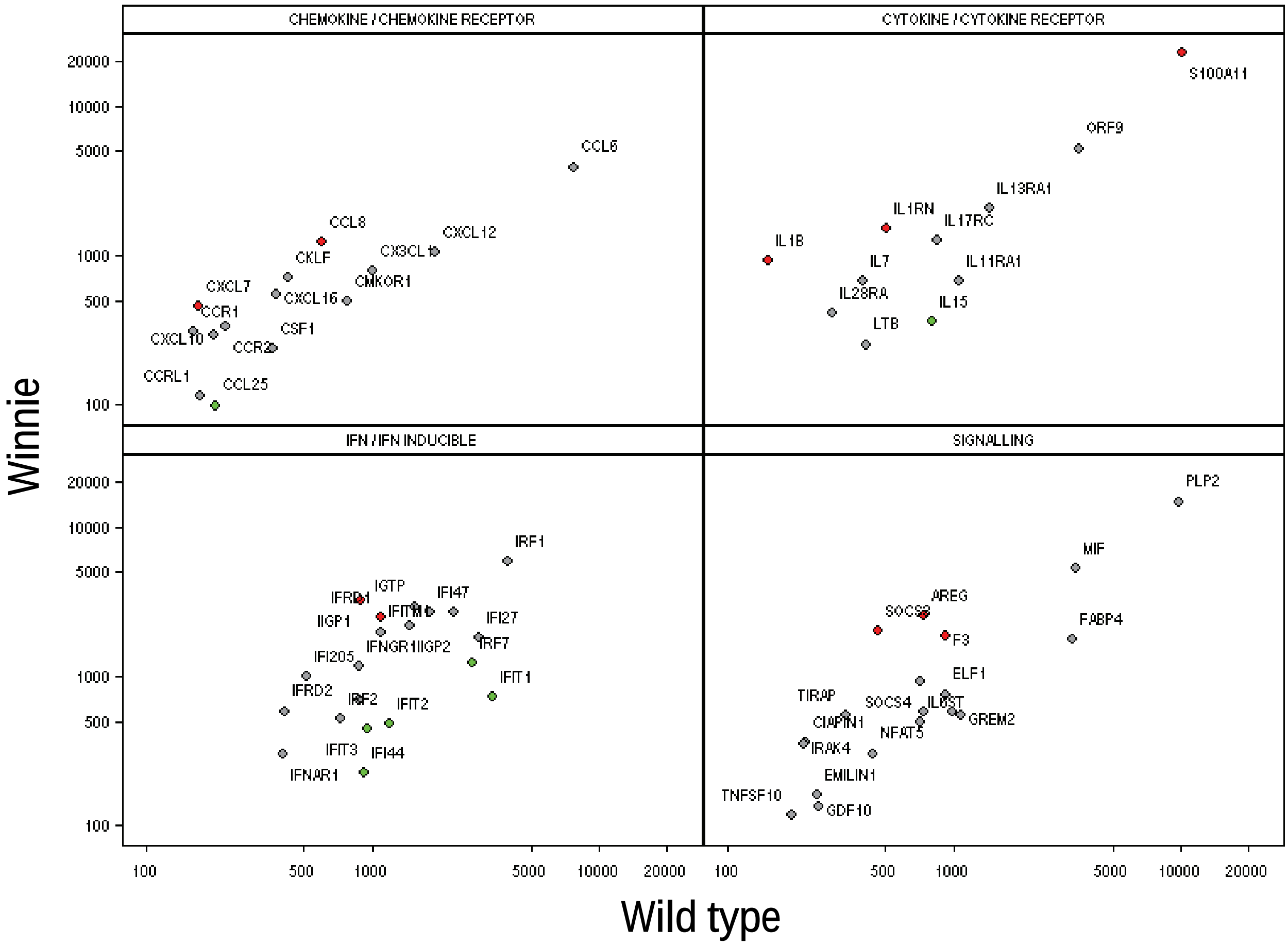
CHEMOKINE / CHEMOKINE RECEPTOR
CYTOKINE / CYTOKINE RECEPTOR
IFN / IFN INDUCIBLE
SIGNALLING
Winnie
Wild type
100
500
1000
5000
10000
20000
CCL6
CCL8
CXCL12
CKLF
CX3CL1
CMKOR1
CXCL7
CXCL16
CCR1
CSF1
CXCL10
CCR2
CCRL1
CCL25
S100A11
ORF9
IL13RA1
IL1RN
IL17RC
IL1B
IL7
IL11RA1
IL28RA
IL15
LTB
IRF1
IGTP
IFI47
IFRD1
IFITM1
IIGP1
IFI27
IFNGR1
IIGP2
IRF7
IFI205
IFIT1
IFRD2
IRF2
IFIT2
IFIT3
IFI44
IFNAR1
PLP2
MIF
AREG
SOCS3
FABP4
F3
ELF1
TIRAP
SOCS4
IL6ST
CIAPIN1
GREM2
NFAT5
IRAK4
EMILIN1
TNFSF10
GDF10
