# Supplementary material for: Aberrant Mucin Assembly in Mice Causes Endoplasmic Reticulum Stress and Spontaneous Inflammation Resembling Ulcerative Colitis
Source: PLoS Med. 2008 Mar 4;5(3):e54. doi: 10.1371/journal.pmed.0050054 (PMC2270292; doi:10.1371/journal.pmed.0050054)
Supplement: Supplementary file 7 [file pmed.0050054.sg007.pdf]

Figure S7

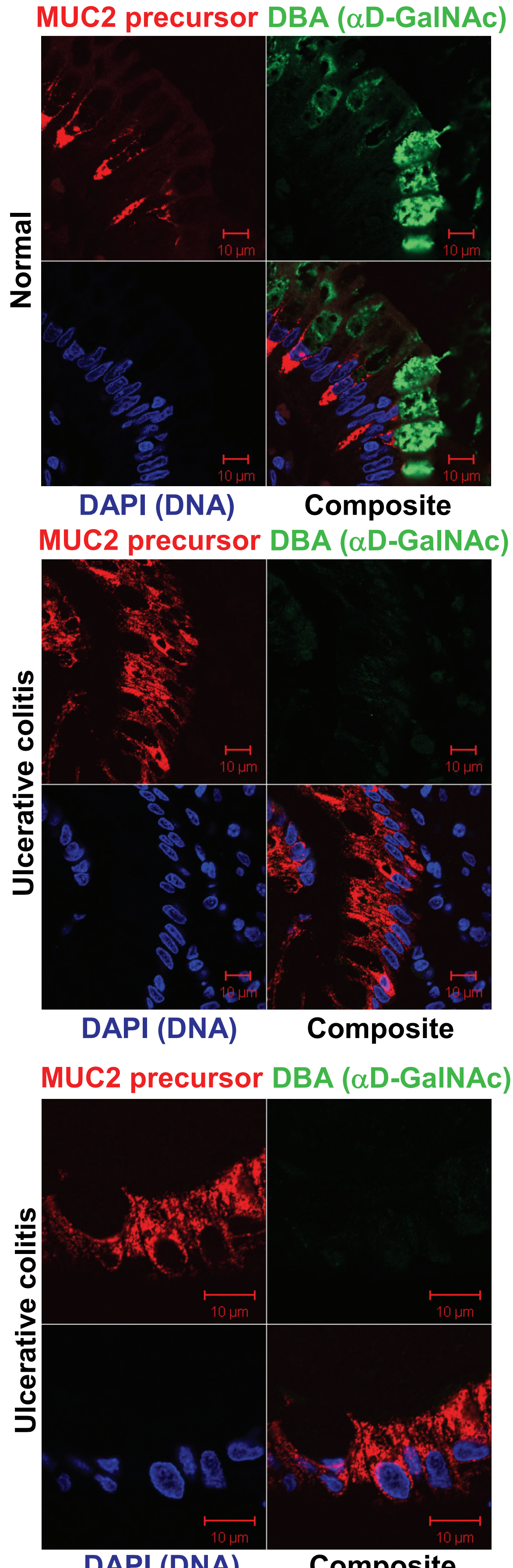

MUC2 precursor DBA (αD-GalNAc)
Normal
10 µm
DAPI (DNA)
Composite
MUC2 precursor DBA (αD-GalNAc)
Ulcerative colitis
10 µm
DAPI (DNA)
Composite
MUC2 precursor DBA (αD-GalNAc)
Ulcerative colitis
10 µm
DAPI (DNA)
Composite
