# Supplementary material for: Aberrant Mucin Assembly in Mice Causes Endoplasmic Reticulum Stress and Spontaneous Inflammation Resembling Ulcerative Colitis
Source: PLoS Med. 2008 Mar 4;5(3):e54. doi: 10.1371/journal.pmed.0050054 (PMC2270292; doi:10.1371/journal.pmed.0050054)
Supplement: Supplementary file 8 [file pmed.0050054.sg008.pdf]

Figure S8

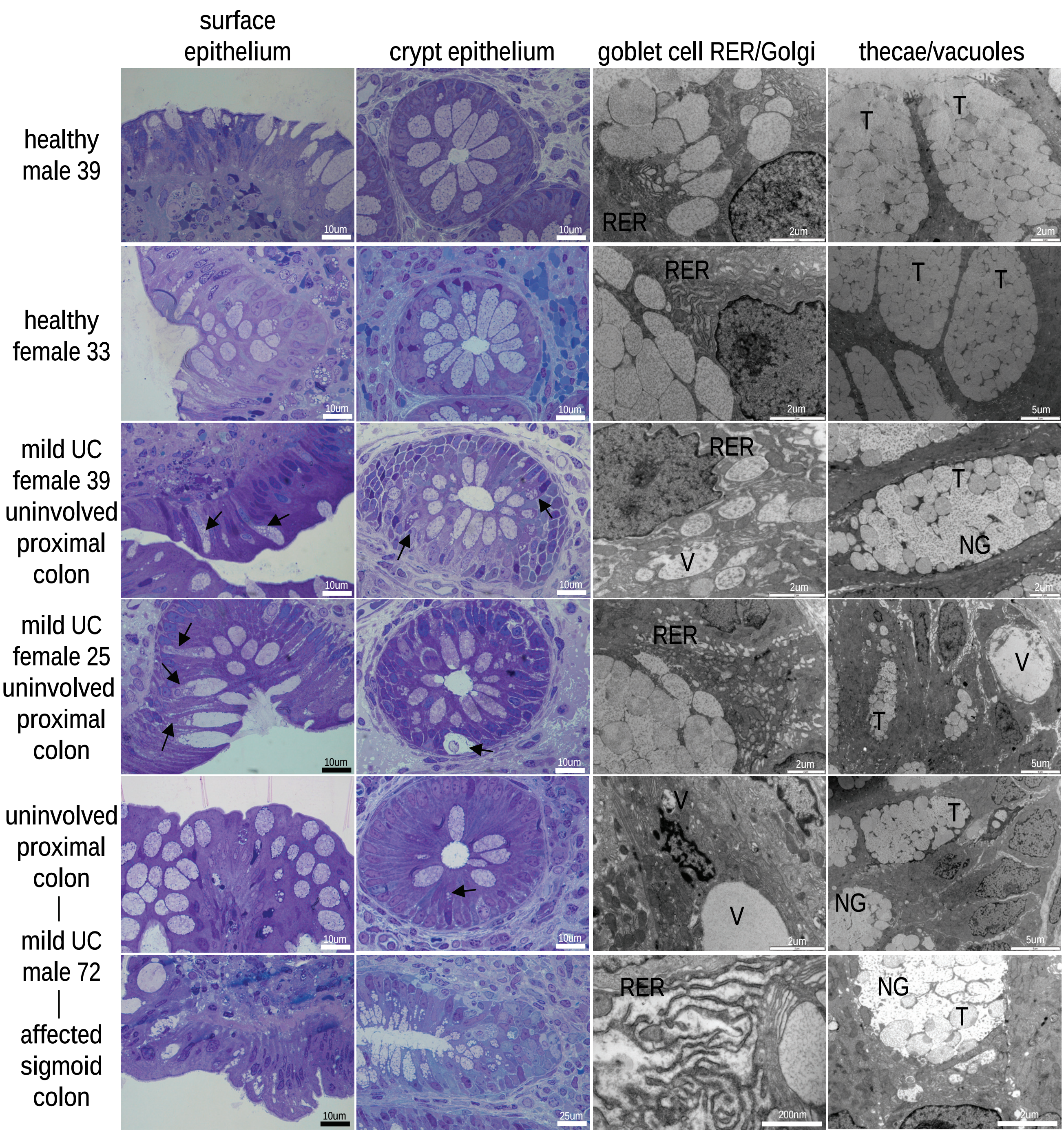

surface epithelium
crypt epithelium
goblet cell RER/Golgi
thecae/vacuoles
healthy male 39
healthy female 33
mild UC female 39 uninvolved proximal colon
mild UC female 25 uninvolved proximal colon
uninvolved proximal colon | mild UC male 72 | affected sigmoid colon
RER
T
V
NG
10um
25um
2um
5um
200nm
