# Supplementary material for: Aberrant Mucin Assembly in Mice Causes Endoplasmic Reticulum Stress and Spontaneous Inflammation Resembling Ulcerative Colitis
Source: PLoS Med. 2008 Mar 4;5(3):e54. doi: 10.1371/journal.pmed.0050054 (PMC2270292; doi:10.1371/journal.pmed.0050054)
Supplement: Supplementary file 9 [file pmed.0050054.st001.doc]

**Table S1.** Histological scoring of murine colitis.

| **A. Large Intestine Inflammation and Damage Scores** | | | |
| --- | --- | --- | --- |
| **Feature** | **Score** | **Description** | |
| Crypt architecture | 0 | Normal architecture | |
|  | 1 | Irregular architecture | |
|  | 2 | Moderate crypt loss (10-50%) | |
|  | 3 | Severe crypt loss (50-90%) | |
|  | 4 | Small-medium size ulcers (10 crypt widths) | |
|  | 5 | Large ulcers (more than 10 crypt widths) | |
| Crypt Length | Caecum | 0 = < 130 uM, 1 = 130-150 um, 2 = 150-200, 3 = 200-250, 4 = >250 | |
|  | Proximal Colon | 0 = < 150 uM, 1 = 150-200 um, 2 = 200-250, 3 = 250-300, 4 = >300 | |
|  | Distal Colon | 0 = < 150 uM, 1 = 150-200 um, 2 = 200-250, 3 = 250-300, 4 = >300 | |
| Crypt abscesses | 0 | No crypt abscesses | |
|  | 1 | 1-5 | |
|  | 2 | 6-10 | |
|  | 3 | >10 | |
| Tissue Damage | 0 | No damage | |
|  | 1 | Discrete lesion | |
|  | 2 | Mucosal erosion | |
|  | 3 | Extensive mucosal damage | |
| Goblet Cell Loss | 0 | <10% loss | |
|  | 1 | 10-25% | |
|  | 2 | 25-50% | |
|  | 3 | >50% | |
| Inflammatory cell infiltration | 0 | Normal lamina propria infiltration | |
|  | 1 | Increasing leukocytes in lamina propria | |
|  | 2 | Confluence of leukocytes extending to sub-mucosa | |
|  | 3 | Transmural extension of inflammatory infiltrate | |
| Lamina propria neutrophils (PMN) per high power field (scored from 10 fields) | 0 | 0-5 PMNs | |
|  | 1 | 6-10 PMNs | |
|  | 2 | 11-20 PMNs | |
|  | 3 | > 20 PMNs | |
| **B. Small Intestinal Damage Inflammation Scores** | | | |
| Crypt and villous architecture | 0 | | Normal architecture |
|  | 1 | | Irregular architecture |
|  | 2 | | Shortening of villi |
|  | 3 | | Some villi completely lost, crypt length increased |
| Paneth Cell Expansion | 0 | | Normal |
|  | 1 | | Mild increase in granulation of Paneth cells |
|  | 2 | | Increase in Paneth cell number and granulation |
| Inflammatory cell infiltration | 0 | | Occasional infiltration |
|  | 1 | | Increasing leukocytes in lamina propria |
|  | 2 | | Prominent leukocytes in villi and between glands |
|  | 3 | | Transmural extension of inflammatory infiltrate |
