# Supplementary material for: Aberrant Mucin Assembly in Mice Causes Endoplasmic Reticulum Stress and Spontaneous Inflammation Resembling Ulcerative Colitis
Source: PLoS Med. 2008 Mar 4;5(3):e54. doi: 10.1371/journal.pmed.0050054 (PMC2270292; doi:10.1371/journal.pmed.0050054)
Supplement: Supplementary file 10 [file pmed.0050054.st002.doc]

**Table S2.** Comparison of the intestinal transcriptome of C57BL/6, *Winnie* and *Eeyore* mice – genes involved in ER stress, anti-

microbial defence, wound repair, epithelial growth, cell cycle and apoptosis.

| **Functional group** | **Gene Name** | **Entrez Gene ID** | **Function** | **Mean ± SD**  **fold difference**  ***Winnie*** | **Mean ± SD fold difference**  ***Eeyore*** |
| --- | --- | --- | --- | --- | --- |
| ER stress, protein degradation, Ca^2+^ metabolism and signaling | GRP78 | 14828 | ER chaperone, regulator of UPR | ↑ 2.2 ± 0.2 | ↑ 2.4 ± 0.1 |
|  | GRP94 | 22027 | ER chaperone | ↑ 1.8 ± 0.2 | ↑ 2.3 ± 0.1 |
|  | Sec61, γ subunit | 53421 | ER pore, removal of misfolded proteins | ↑ 2.2 ± 0.3 | ↑ 2.1 ± 0.1 |
|  | Peptidyl prolyl isomerase H | 624822 | Protein folding | ↑ 2.3 ± 0.6 | ↑ 2.1 ± 0.2 |
|  | Ubiquitin-specific peptidase 12 | 219333 | Protein degradation | ↑ 2.1 ± 0.6 | ↑ 2.0 ± 0.2 |
|  | Ubiquitin-conjugating enzyme E2C | 68612 | Protein degradation | ↑ 2.4 ± 0.5 | ↑ 2.7 ± 0.3 |
|  | Phospholamban | 18821 | Negative regulator of SERCA Ca^2+^ pump | ↓ 4.2 ± 1.8 | ↓ 5.2 ± 2.2 |
|  | Calcium-calmodulin dependent protein kinase 1D | 227541 | Ca^2+^ signaling | ↓ 3.1 ± 0.6 | ↓ 4.1 ± 1.7 |
| Anti-microbial,  wound repair | SLPI | 20568 | Protease inhibitor, anti-microbial, mucosal defense | ↑ 18.5 ± 16.9 | ↑ 22.6 ± 6.3 |
|  | Lipocalin 2 | 16819 | anti-microbial/wound repair | ↑ - NE in wt | ↑ - NE in wt |
| Wound repair, epithelial growth and cell cycle | Amphiregulin | 11839 | Growth factor, EGF receptor ligand | ↑ 3.5 ± 0.7 | ↑ 2.8 ± 0.6 |
|  | Epiregulin | 13874 | Growth factor, EGF receptor ligand | ↑ 2.8 ± 1.1 | ↑ 3.1 ± 1.5 |
|  | Heparin-binding epidermal growth factor | 15200 | Growth factor, EGF receptor ligand | ↑ 2.9 ± 0.6 | ↑ 2.1 ± 0.1 |
|  | Stratifin | 55948 | Growth, cell cycle progression | ↑ 4.4 ± 0.9 | ↑ 4.7 ± 1.5 |
|  | Receptor for hyaluronan mediated motility (RHAMM) | 15366 | wound healing | ↑ 2.7 ± 0.4 | ↑ 2.1 ± 0.5 |
|  | Cyclin B1 | 268697 | Cell cycle regulation | ↑ 2.5 ± 0.4 | ↑ 2.1 ± 0.7 |
|  | Cyclin-dependent kinase inhibitor 1A (p21) | 12575 | Cell cycle regulation, apoptosis | ↑ 2.8 ± 0.4 | ↑ 2.6 ± 0.4 |
|  | Protein tyrosine kinase 6 (PTK6) | 20459 | Cell cycle exit and differentiation | ↑ 4.3 ± 1.8 | ↑ 4.1 ± 1.0 |
| Apoptosis | Bcl-2 | 12043 | Apoptosis inhibitor | ↓ 2.3 ± 0.8 | ↓ 2.7 ± 0.4 |
|  | Intectin | 67038 | Intestinal apoptosis promoter | ↑ 5.1 ± 1.1 | ↑ 5.3 ± 3.2 |
|  | RIP3 kinase | 56532 | Apoptotic signaling and NF-KB activation | ↑ 3.3 ± 0.8 | ↑ 3.6 ± 0.6 |

Intestinal RNA was analysed as per Materials and Methods using Affymetrix Mouse 430_2 olignucleotide arrays. Mean ± SD of gene expression in *Winnie* and *Eeyore* relative to the mean expression in wild types are shown (N = 3 per group). A subset of genes with either increased (↑) or decreased (↓) expression in both *Winnie* and *Eeyore* are shown in functional groups relevant to goblet cell stress and the development of inflammation. The entire microarray data set can be accessed at NCBI GEO Accession No. GSE9913. NE in wt = not expressed in wild type (fold change is not assessable).
