# Supplementary material for: Aberrant Mucin Assembly in Mice Causes Endoplasmic Reticulum Stress and Spontaneous Inflammation Resembling Ulcerative Colitis
Source: PLoS Med. 2008 Mar 4;5(3):e54. doi: 10.1371/journal.pmed.0050054 (PMC2270292; doi:10.1371/journal.pmed.0050054)
Supplement: Supplementary file 11 [file pmed.0050054.st003.doc]

**Table S3.** Comparison of the intestinal transcriptome of C57BL/6, *Winnie* and *Eeyore* mice – genes involved in inflammation,

metabolism, detoxification and the mucus barrier.

| **Functional group** | **Gene Name** | **Entrez Gene ID** | **Function** | **Fold difference**  ***Winnie* Mean ± SD** | **Fold difference**  ***Eeyore* Mean ± SD** |
| --- | --- | --- | --- | --- | --- |
| Inflammation | IL-1β | 16176 | Inflammatory cytokine | ↑ 6.1 ± 5.2 | ↑ 7.5 ± 1.5 |
|  | IL-1β receptor antagonist | 16181 | Inflammatory response | ↑ 3.6 ± 0.9 | ↑ 2.7 ± 0.8 |
|  | SOCS3 | 12702 | Modulator of cytokine signaling | ↑ 4.4 ± 2.0 | ↑ 3.6 ± 0.6 |
|  | Immunoglobulin heavy chain 1a (serum IgG2a) | 380793 | Humoral immunity | ↑ 1.7 ± 1.0 | ↑ 2.0 ± 1.2 |
|  | Immunoglobulin heavy chain (V7183 family) | 16059 | Humoral immunity | ↑ 2.4 ± 1.3 | ↑ 2.5 ± 1.5 |
|  | Guanine-nucleotide binding protein 2 | 14469 | IFN-g mediated GTPase | ↑ 3.6 ± 1.0 | ↑ 4.6 ± 3.5 |
|  | CD14 antigen | 12475 | Cell surface co-receptor for LPS | ↑ 2.0 ± 0.6 | ↑ 2.1 ± 0.4 |
|  | S-100 calcium binding protein A14 | 66166 | Macrophage activation | ↑ 2.8 ± 0.9 | ↑ 2.6 ± 0.2 |
|  | S-100 calcium binding protein A16 | 67860 | Macrophage activation | ↑ 2.8 ± 0.6 | ↑ 2.4 ± 0.3 |
|  | Endothelin-2 | 13615 | Inflammatory modulator, vasoconstriction | ↓ 3.3 ± 1.7 | ↓ 4.0 ± 1.5 |
|  | chemokine (C-X-C motif) ligand 9 (cxcl9) | 17329 | Inflammatory chemokine | ↑ - NE in wt | ↑ - NE in wt |
|  | Restin-like molecule β | 21123 | Goblet cell inflammatory modulator | ↓ 6.5 ± 1.0 | ↑ 1.7 ± 0.6 |
| Metabolism, detoxification | PPAR alpha | 19013 | fatty acid metabolism | ↓ 2.3 ± 0.9 | ↓ 2.2 ± 0.0 |
|  | phosphoenolpyruvate carboxykinase 1 | 18534 | Gluconeogenesis | ↓ 3.5 ± 1.0 | ↓ 3.9 ± 1.2 |
|  | 3-hydroxy-3-methylglutaryl-Coenzyme A synthase 2 | 15360 | Cholesterol synthesis | ↓ 3.2 ± 1.8 | ↓ 3.0 ± 1.7 |
|  | carbonic anhydrase 8 | 12319 | Detoxification enzyme | ↓ 4.5 ± 1.4 | ↓ 4.4 ± 1.2 |
|  | glutathione S-transferase, alpha 1 (Ya) | 14857 | Xenobiotic metabolizing enzyme | ↑ 4.7 ± 1.3 | ↑ 3.4 ± 0.1 |
| Mucus barrier | Muc2 | 17831 | Secreted mucin glycoprotein | ↓ 2.3 ± 0.4 | ↓ 2.3 ± 0.3 |
|  | UDP-GlcNAc:betaGal beta-1,3-N-acetylglucosaminyltransferase 6 (core 3 synthase) | RIKEN cDNA 6030413G23 | Mucin glycotransferase | ↓ 5.6 ± 0.6 | ↓ 5.2 ± 0.5 |
|  | UDP-N-acetyl-alpha-D-galactosamine: polypeptide N-acetylgalactosaminyltransferase 7 | 108150 | Mucin glycotransferase | ↓ 4.0 ± 2.2 | ↓ 2.4 ± 0.4 |

Intestinal RNA was analysed as per Materials and Methods using Affymetrix Mouse 430_2 olignucleotide arrays. Mean ± SD of gene expression in *Winnie* and *Eeyore* relative to the mean expression in wild types are shown (N = 3 per group). A subset of genes with either increased (↑) or decreased (↓) expression in both *Winnie* and *Eeyore* are shown in functional groups relevant to goblet cell stress and the development of inflammation. The entire microarray data set can be accessed at NCBI GEO Accession No. GSE9913. NE in wt = not expressed in wild type (fold change is not assessable).
